# Rictor promotes cell migration and actin polymerization through regulating ABLIM1 phosphorylation in Hepatocellular Carcinoma

**DOI:** 10.7150/ijbs.46285

**Published:** 2020-09-01

**Authors:** Xin Dong, Mei Feng, Hui Yang, Hengkang Liu, Hua Guo, Xianshu Gao, Yucun Liu, Rong Liu, Ning Zhang, Ruibing Chen, Ruirui Kong

**Affiliations:** 1 Translational Cancer Research Center, Peking University First Hospital, Beijing. 100034, P.R. China.; 2 Department of General Surgery, Peking University First Hospital, Beijing. 100034, P.R. China.; 3 Department of Radiation Oncology, Peking University First Hospital, Beijing. 100034, P.R. China.; 4 School of Pharmaceutical Science and Technology, Tianjin University, Tianjin, 300072, P.R. China.; 5 Laboratory of Tumor Cell Biology, Tianjin Medical University Cancer Institute and Hospital, Tianjin, 300060, P.R. China.

**Keywords:** hepatocellular carcinoma, Rictor, ABLIM1, phosphorylation, actin polymerization

## Abstract

As one of the most ominous malignancies, hepatocellular carcinoma (HCC) is frequently diagnosed at an advanced stage, owing to its aggressive invasion and metastatic spread. Emerging evidence has demonstrated that Rictor, as a unique component of the mTORC2, plays a role in cell migration, as it is dysregulated in various cancers, including HCC. However, the underlying molecular mechanism has not been well-characterized. Here, evaluation on a tissue-array panel and bioinformatics analysis revealed that Rictor is highly expressed in HCC tissues. Moreover, increased Rictor expression predicts poor survival of HCC patients. Rictor knockdown significantly suppressed cell migration and actin polymerization, thereby leading to decreased nuclear accumulation of MKL1 and subsequent inactivation of SRF/MKL1-dependent gene transcription, i.e. Arp3 and c-Fos. Mechanistically, we identified ABLIM1 as a previously unknown phosphorylation target of Rictor. Rictor interacts with ABLIM1 and regulates its serine phosphorylation in HCC cells. We generated ABLIM1 knockout cell lines of HCC, in which dominant negative mutations of Ser 214 and Ser 431 residues inhibited the ABLIM1-mediated actin polymerization and the MKL1 signaling pathway. Overall, ABLIM1 phosphorylation induced by Rictor plays an important role in controlling actin polymerization in HCC cells.

## Introduction

Hepatocellular carcinoma (HCC) is a fatal malignancy with high incidence and morbidity, accounting for the second most cancer-related deaths worldwide 1-4. Although the treatment for HCC have been developed, the prognosis of HCC remains an overall 5-year survival rate of ~30% 5,6. For advanced patients, the survival time is even shorter because of tumor cell invasion and metastatic spread to other distal organs 7-9. However, the underlying mechanism regulating cell invasion and metastasis remains elusive.

Mammalian target of rapamycin (mTOR) is a serine/threonine kinase that regulates diverse biological processes, including cell growth, survival, metabolism, autophagy and immunity, by responding to growth factors or cellular energy 10-13. mTOR is usually assembled into several multiprotein complexes, such as mTOR complex 1 (mTORC1) and mTOR complex 2 (mTORC2) 14-16. Accumulating evidence has indicated that mTORC2 activity is frequently dysregulated in a wide variety of cancers 17-21. Rapamycin insensitive companion of mTOR (Rictor) is a unique subunit of mTORC2 and is required for mTORC2 activity. It has been reported that Rictor is aberrantly expressed in majority of human cancers, including HCC 22-24. Some studies have demonstrated that Rictor regulates the activation of multiple targets, such as AKT, SGK and PKC, which control actin reorganization, cell growth and cell migration 25-31. Nonetheless, the role of Rictor in HCC and its precise regulatory mechanisms have not been well-characterized.

To dissect the Rictor signaling pathway, we performed quantitative phosphoproteomics analysis and identified ABLIM1 as a potential substrate for Rictor. ABLIM1 (actin-binding LIM protein 1) was originally characterized as a dematin-like protein in the retinal tissues 32. It can bind to actin filaments and mediate the dynamics of actin-associated membrane skeletal rearrangement 32-35. Several studies have shown that ABLIM1 functions through phosphorylation modification in distinct tissues. For instance, ABLIM1 is highly phosphorylated in the retina under light-adapted conditions 32 and is phosphorylated by DYRK1A kinase when it was involved in Hh signaling pathway 34. However, the underlying molecular mechanism of how ABLIM1 is modulated by phosphorylation, thereby organizing coordinated changes in the actin cytoskeleton to influence cell migration, remains largely unknown.

Here, we report that Rictor contributes to cell migration and actin polymerization through interacting with and phosphorylating endogenous ABLIM1 in HCC cells. In ABLIM1 knockout cells, the negative dominant mutants of ABLIM1 at Ser 214 and Ser 431 residues impaired the ABLIM1-regulated actin polymerization, the nuclear distribution of MKL1 and the activation of Arp3. To our knowledge, these data identify ABLIM1 as a novel Rictor phosphorylation target involved in cytoskeletal regulation.

## Materials and Methods

### Cell culture and reagents

The human liver cancer cell line HCCLM3 was obtained from the Liver Cancer Institute, Zhongshan Hospital, Fudan University (Shanghai, China). The human liver cancer cell line Hep3B and the human embryonic kidney cell line HEK293T were obtained from American Type Culture Collection (ATCC). All cells were cultured in Dulbecco's Modified Eagle's medium (DMEM) (Corning) supplemented with 10% fetal bovine serum (FBS) (VISTECH), 100 U/ml of penicillin and 100 µg/ml of streptomycin (P/S) (Hyclone) in a humidified incubator at 37°C with 5% CO_2_. Commercially available antibodies used in this study included anti-Rictor (Bethyl, A500-002A), anti-ABLIM1 (Abcam, ab222824 and Proteintech, 15129-1-AP), anti-MKL1 (Proteintech, 21166-1-AP), anti-phosphoserine (Abcam, ab9332), anti-phospho-AKT (Ser473) (Cell Signaling Technology, 4060T), anti-AKT (Cell Signaling Technology, 4685S), anti-Flag (Sigma, F3165-2MG), anti-β-Actin (Santa Cruz, sc-69879) and anti-GAPDH (Santa Cruz, sc-47724), anti-c-Fos (Cell Signaling Technology, 2250S), anti-Arp3 (Proteintech, 13822-1-AP). Mitomycin C was from Selleck (S8146).

### CRISPR-Cas9-sgRNA plasmid construction

The sequences for sgRNAs targeting ABLIM1 genes were designed using http://crispr.mit.edu/ and shown in Table S1. For construction of CRISPR-Cas9-sgRNA plasmids, annealed oligos against sgRNA were inserted into the pSpCas9(BB)-2A-GFP (PX458) vector at the BbsI (NEB) site. Plasmids were transfected into liver cancer cells using Lipofectamine 3000 reagents (Life Technologies) according to the manufacturer's instructions. After 24 hrs of transfection, a single clonal cell was selected by flow cytometry based on its GFP fluorescence. Cellular genomic DNA was extracted and amplified with primers designed to span the expected indel positions. The PCR products were sequenced by Sanger sequencing. Off-target sites were predicted and analyzed with the T7E1(NEB) assay. All primers for ABLIM1 or the corresponding off-targets are indicated in supplementary Table S1.

### siRNA and construction of Rictor and ABLIM1 plasmids

Three different siRNAs against Rictor or ABLIM1 were synthesized together with a negative control siRNA (siNC) (Shanghai Gene Pharma Co., Ltd). The siRNAs sequences are shown in supplementary Table S1. The SRF/MKL1-binding promoter luciferase reporter (PGL433) and internal control beta-actin Renilla plasmids were gifted from Dr. Ceshi Chen. For full-length Rictor which encodes the 1733-amino acid protein, we designed 5 overlapping fragments, followed by PCR amplification with the corresponding primers. Then, the five segments produced were recombined together by PCR-driven overlap extension. The construct was inserted into the pcDNA3.1-3×Flag vector by a homologous recombination kit (YiSheng). Full-length fragments of wild type ABLIM1 and its mutants (S214D, S214A, S431D, S431A, 2SD and 2SA) were generated by overlap extension PCR and subcloned between the BamHI (NEB) and EcoRI (NEB) sites of pcDNA3.1-Myc or EGFPN1 vectors. Full-length MKL1 was obtained from overlap extension PCR and inserted into the retro-virus PQCXIN vector tagged with mCherry. All constructs were verified by DNA sequencing. Sequences of primers in plasmid construction are also shown in supplementary Table S1.

### Tissue microarray and immunohistochemical analysis

We collected 45 pairs of hepatocellular cancer tissue samples with corresponding matched adjacent non-tumor tissues from Tianjin Medical University Cancer Institute and Hospital. All specimens were obtained with written informed consent of the patients in accordance with institutional and national guidelines. The tissue samples were fixed with formalin and embedded with paraffin, and then sectioned to produce serial 4-µm sections. The tissue microarray was immunostained with the anti-Rictor antibodies, followed by incubation of HRP-conjugated secondary Ab and cover development with DAB. Rictor expression levels were scored by two independent pathologists, blinded to the clinical characteristics of the patients. The scoring system has been described previously 36. In brief, Rictor immunostaining was scored according to the staining intensity and extent. Staining intensity was classified as 0 (negative), 1 (weak), 2 (moderate) and 3 (strong). Staining extent dependent on the percentage of positive tumor cells were divided into 0 (<5%), 1 (5-25%), 2 (26-50%), 3 (51-75%), and 4 (>75%). The final score was determined by multiplying the intensity and the quantity scores, which yielded a range from 0 to 12. Two-tailed Student's t test was used to compare the difference of Rictor expression in HCC and non-tumor tissue samples.

### Real-time PCR

Total RNA from liver cancer cells was isolated using Trizol reagent (Invitrogen). DNA was removed by treatment with RNase-free DNase (TianGen) and cDNAs were prepared with reverse transcriptase with an oligo (dT)18 primer (TianGen). Real-time PCR was carried out using corresponding specific primers indicated in supplementary Table S1. Relative quantitation was determined using the AriaMx Real-Time PCR System (G8830A) that measures real-time SYBR green fluorescence (TianGen). The PCR conditions were as follows: 15 min at 95°C for one cycle followed by 40 cycles of 10 sec at 95°C, 30 sec at 55°C (according to Tm) and 32 sec at 72°C. We calculated gene expression by the comparative Ct method (2-ΔΔCt) with GAPDH as an internal control.

### Immunoprecipitation and Western blotting

Cells were lysed in lysis buffer [50mM Tris-HCl (pH 7.4), 250mM NaCl, 1% NP-40, 2 mM EDTA, 1mM DTT, 10% glycerol, proteinase inhibitors (ROCHE) and phosphatase inhibitors (ROCHE)]. Insoluble materials were pelleted and supernatants were incubated with antibodies for 2 hrs, followed by further incubation with 30 µl of Protein A/G dynabeads (Invitrogen) for another 2 hrs at 4°C. After beads were washed three times with lysis buffer, immunoprecipitated proteins were separated on SDS-PAGE and transferred onto PVDF membranes. The membrane was blocked with 5% nonfat milk and probed using specific primary antibodies, followed by incubation with HRP-conjugated secondary antibodies (Cell Signaling Technology). The bands were detected with an ECL system (Millipore) using a digital detection machine (SYNGENE).

### Proximity ligase assay (PLA)

Cells were fixed with 4% paraformaldehyde (PFA) for 10 min, followed by permeabilization with methanol for 30 min. Cells were incubated in blocking solution for 1 hr at room temperature. After the slides were incubated with mouse anti-human Rictor and rabbit anti-ABLIM1 antibodies, cells were stained with two secondary antibodies linked to PLA probes (one PLUS and one MINUS). The probes hybridized to circular DNA were amplified with fluorescently-labeled oligonucleotide. The spots of proximity were visualized by fluorescence microscopy (OLYMPUS).

### Immunofluorescence

Cells were fixed with 4% paraformaldehyde (PFA) for 20 min and blocked with blocking buffer (5% NGS, 0.3% TritonX100 in PBS) for 1 hr at room temperature. Cells were subsequently incubated with anti-Rictor and anti-ABLIM1 antibodies at 4°C overnight. After washing with PBS three times, cells were stained with secondary antibodies conjugated to Alexa488 (ZSGB-BIO), Alexa594 (ZSGB-BIO) or Alexa594-labeled phalloidin (Invitrogen) at 37°C for 1 hr. Images were obtained by confocal microscopy (OLYMPUS) and further processed and analyzed with ImageJ software.

### Dual luciferase assay

The SRF/MKL1-binding promoter luciferase reporter has been described previously 37. After HEK293T cells were seeded in 12-well plates, the cells were cotransfected with the SRF/MKL1-binding promoter luciferase reporter and internal control beta-actin Renilla plasmids in triplicate. One day after transfection, luciferase activities were measured using the dual luciferase reporter assay system (Promega Corp).

### Transwell

Cells (1.5 × 10^5^) were suspended in medium containing 0.1% BSA and seed in transwell insert with 8 µm pore polycarbonate filter membranes. Medium with 10% FBS were added into lower chamber of transwell for inducing cell migration through pores. After incubation for 24 hrs, cells were fixed by methanol and stained with 0.1% crystal violet. The migrated cells were photographed with microscopy and six fields were randomly chosen to count the cell number with ImageJ software. All samples were tested in triplicate and the data were presented as the mean ± SEM.

### Wound healing assay

After cells were seeded onto fibronectin (R&D)-coated coverslips and grown to confluence, a scratch was made with a sterile micropipette tip to form a wound area. Cells were rinsed extensively to remove the dislodged cells with culture media. Then, cells were cultured in media containing Mitomycin C (5 µM) for 48 hrs. Cell migration was monitored by microscopy, and the images were taken at 0 hr and 48 hrs. The forward distance of cell migration from the line of wound formation was measured by ImageJ software.

### Cell cycle assay

For DNA content analysis, cells were trypsinized and washed with PBS, and fixed in 75% ice-cold ethanol at 4°C overnight. Cells were rehydrated in PBS on the second day. Following RNase digestion, cells were stained with propidium iodide (PI). Flow cytometry analysis was performed using red (PI) emission (at 630 nm). The data from 10^4^ cells were collected and analyzed by using FlowJo software.

### Cell counting kit-8

A growth curve was plotted with the Cell Counting Kit-8 (CCK-8) (Bestbio) according to the manufacturer's instructions. In brief, cells were seeded into 96-well plates, and CCK-8 was added to these cells and incubated for 1 hr at different time points. Absorbance at 450 nm was measured using a microplate reader. The experiment was repeated three times in duplicate. Growth curves were plotted using the mean±SEM of absorbance versus the time points.

### Microarray data analysis

The UALCAN and Oncomine databases were used to analyze mRNA levels of Rictor or ABLIM1 in liver cancer and control samples. The cBioPortal dataset was used to analyze genetic alterations of Rictor or ABLIM1 in liver cancer tissue samples. For patient survival analysis, the association between Rictor or ABLIM1 expression and prognosis in liver cancer patients was analyzed with GEPIA, followed by log-rank testing for significance. The gene expression correlation of Ritor or ABLIM1 and Arp3 or c-Fos was analyzed with TCGA dataset.

### Statistical analysis

All statistical calculations were carried out using GraphPad Prism 7 software. Two-tailed Student's *t*-test, one-way ANOVA, two-way ANOVA and chi-square tests were used to determine the statistical significance of differences between two groups and among more than two groups, respectively. Statistical significance was determined as *p* < 0.05. Error bars represent the standard error of the mean.

## Results

### Increased Rictor expression in HCC tissues positively correlates with poor prognosis of patients

**Figure 1:**
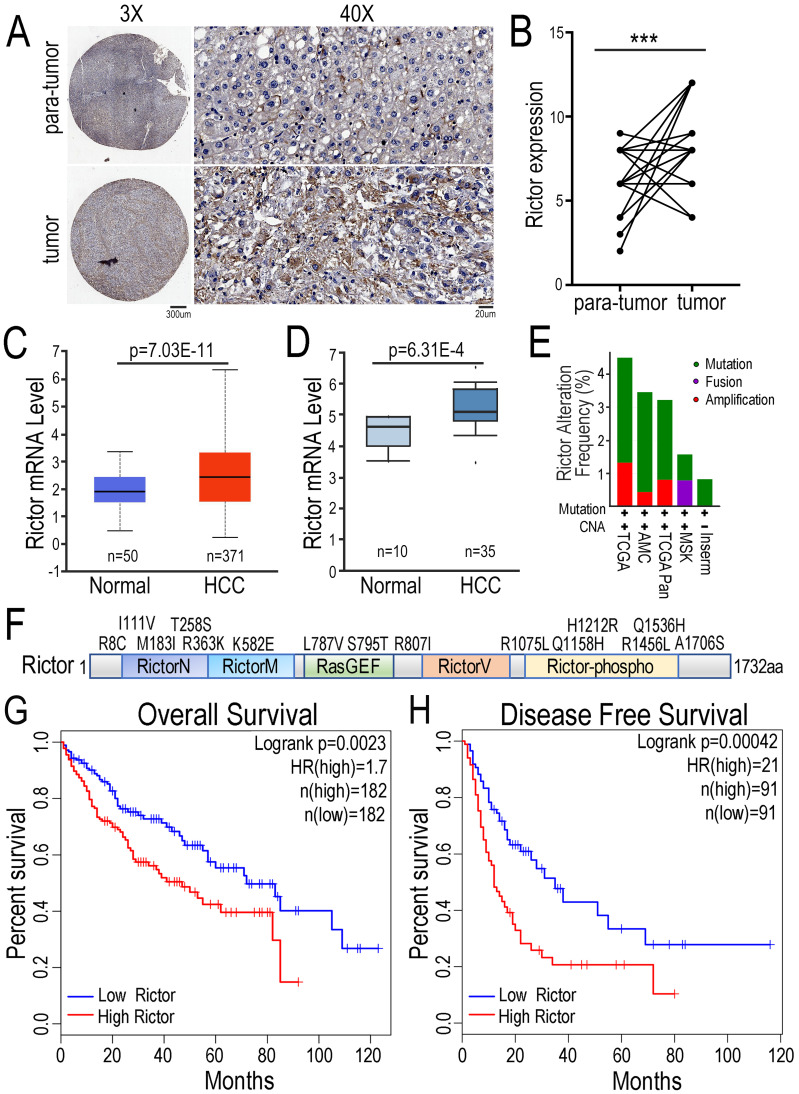
** Expression of Rictor in HCC is positively associated with poor prognosis of patients.** (**A**) Immunohistochemistry was carried out to detect Rictor protein in 45 pairs of human HCC and matched adjacent non-tumor tissue samples. Representative images of Rictor immunostaining on tissue microarrays are shown at low (×3) and high (×40) magnification. Scale bars: 300 µm and 20 µm. (**B**) The IHC scores between HCC and non-cancerous tissues were quantified using two-tailed Student's t-test. (**C-D**) The expression of Rictor was analyzed in HCC and normal hepatocellular tissues from the UALCAN and Oncomine database. Rictor mRNA levels are expressed as log2 median-centered intensity. P-values were determined by Student's *t*-test. (**E**) The data showed genetic alteration frequency of Rictor gene from the cBioPortal dataset, containing copy number alterations (CNA) and mutation, fusion and amplification. (**F**) The schematic drawing depicts the mutation distribution of Rictor across protein domains in liver carcinoma. (**G-H**) The Kaplan-Meier plots of overall survival and disease-free survival from the GEPIA database divided by median levels of Rictor expression in HCC, with hazard rate (HR) and log-rank test p-values displayed.

To explore the function of Rictor in HCC pathogenesis, we performed the immunohistochemical (IHC) assay with tissue-array panel containing 45 pairs of HCC tissues and matched adjacent non-tumor liver tissues. We found that Rictor expression was significantly increased in HCC tissue samples compared with para-tumor tissue controls (Figure 1A, B). To support our findings, we statistically analyzed the expression of Rictor in HCC tissues in two databases. The data showed that Rictor was highly expressed in HCC samples, compared with the control normal liver tissues, consistent with our IHC data (Figure 1C, D). Next, the gene alterations of Rictor in the liver cancer samples were analyzed using different datasets in the cBioPortal database. We found that the *RICTOR* gene was genetically altered in approximately 1~4% of human liver cancer cases, including mutation, fusion or amplification (Figure. 1E). Notably, multiple somatic mutations of Rictor gene across its protein domains in liver cancer are indicated in Figure 1F. To further evaluate the contribution of Rictor in prognosis for HCC patients, we utilized another liver cancer microarray from the GEPIA dataset, in which patients were stratified into two groups according to Rictor expression in these tumors. Kaplan-Meier analysis revealed that HCC patients with higher expression of Rictor displayed significantly shorter overall survival (OS) and disease-free survival (DFS) (Figure 1G, H). Collectively, these data suggest the potential oncogenic properties of Rictor in HCC and the clinical significance of Rictor as a promising prognostic indicator of OS and DFS for HCC patients.

### Rictor regulates HCC cell migration and actin polymerization

**Figure 2:**
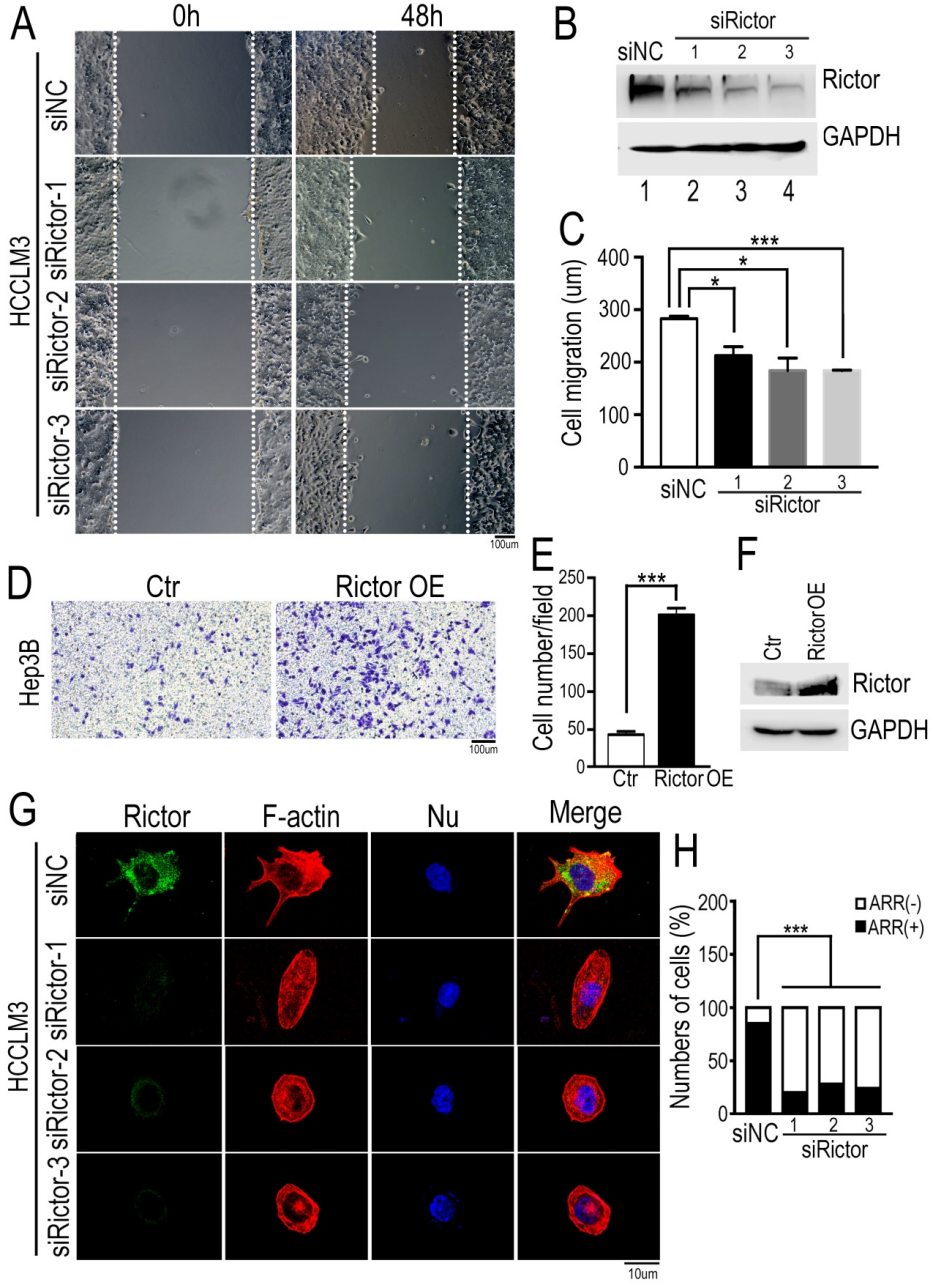
** Knockdown of Rictor inhibits HCC cell migration and actin polymerization.** (**A**) Three Rictor-specific siRNAs (siRictors) or negative control (siNC) were transfected into HCCLM3 cells for 48 hrs, and cells were plated onto the coverslips coated with fibronectin (50 µg/ml). Wound scratches were formed with a 10 µl pipette tip and the cells migrated toward the center of the wound area from two edges marked with the white dotted lines in the presence of media supplemented with mitomycin C (5 uM), which inhibits cell proliferation. Representative images taken at indicated times are shown (Scale bar: 100 µm). (**B**) Rictor expression was detected at 72 hrs post-transfection by Western blotting. Three siRNAs specifically suppressed the expression of endogenous Rictor. GAPDH was used as an internal control. (**C**) Cell migration distance of distinct group was measured and the difference was quantified using the two-tailed unpaired *t*-test. The results were summarized from three independent experiments. (**D**) Hep3B cells transfected with control or a plasmid expressing Rictor were suspended in 200 µl DMEM medium containing 0.1% BSA and then seed upper chambers. After 24 hrs of incubation, the migrated cells were fixed with methanol, followed by staining with 0.1% crystal violet. The migrated cells were photographed with microscopy. (**E**) Six fields were randomly chosen to count the cell number with ImageJ software. The difference of cell number between two groups was quantified using two-tailed Student's *t*-test. The results were summarized from three independent experiments. (**F**) The protein levels of Rictor were detected by Western blotting using specific anti-Rictor antibodies. GAPDH was utilized as an internal control. (**G**) Cells transfected with siRictors or siNC were seeded onto the coverslips coated with fibronectin and then stained with Alexa594-conjugated phalloidin, followed by DAPI staining for nuclear staining. Representative images were visualized with a microscope and the actin-rich regions (ARR) of cells from each group were quantified, including filopodia, lamellipodia and membrane ruffles (Scale bar: 10 µm). (**H**) The data were analyzed by the chi-square test. n≥50.

To assess the effect of Rictor on HCC cell migration, we established a wound healing assay using the HCCLM3 cells (Figure 2A). The cells were transfected with three distinct siRNAs specifically against Rictor (siRictors) or a negative control (siNC). Immunoblotting experiments confirmed that siRictors were efficient for inhibiting the Rictor expression in HCC cells (Figure 2B). After Rictor-deficient HCCLM3 cells were grown to confluence on fibronectin-coated coverslips, a wound was introduced with a fine tip. The cells migrated from the wound edge line marked by a white dotted line at the zero time point. Then, time-lapse imaging was conducted to monitor cell migration at the different points as indicated (Figure 2A, C). The results demonstrated that Rictor knockdown (KD) significantly suppressed the migration of HCCLM3 cells.

To further confirm our findings, we performed the transwell assay using another HCC cells, Hep3B. The cells overexpressing Rictor were seeded a well with filter membrane, through which cells migrated the opposite. Contrary to our data from wound healing assay, we found that ectopic expression of Rictor significantly promotes cell migration (Figure 2D-F). Collectively, these results indicate that Rictor is capable of regulating cell migration.

To test if Rictor plays a role in cell growth, we performed the colony formation assay in Rictor-KD HCCLM3 cells. Both the size and the number of colonies were examined and a growth curve was plotted. As shown in Supplementary Figure S1A and B, we observed no difference in cell proliferation between control and Rictor-KD cells. Subsequently, the cell cycle was analyzed by flow cytometry when Rictor was silenced. The results showed no significant alterations in G1, S or G2/M phases in control and Rictor-KD cells, suggesting that Rictor does not affect the cell cycle in liver cancer cells (Supplementary Figure S1C, D). Overall, Rictor regulates HCC cell migration, but does not affect cell growth.

Furthermore, to understand whether Rictor is responsible for actin polymerization in HCC cells, we stained for F-actin in fixed Rictor-KD HCCLM3 cells using fluorescently labeled phalloidin. Conventional wide field fluorescence images of control cells displayed F-actin enrichment in peripheral ruffles characteristic of filopodia. In contrast, Rictor-KD cells did not generate such sheet-like membrane protrusions (Figure 2G, H). Intriguingly, we found that Rictor was localized at the cell protrusion shown at the top of Figure 2G. These data indicate that Rictor promotes protrusion formation of HCC cells.

**Figure 3:**
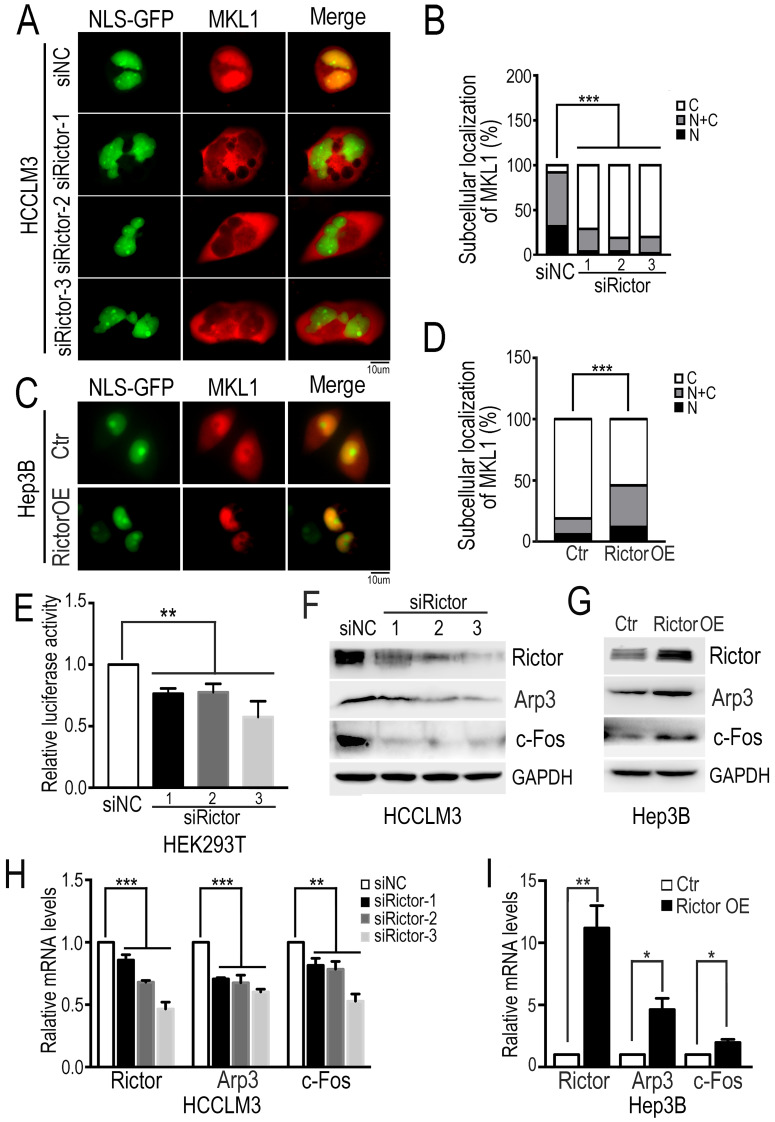
** Rictor knockdown suppresses MKL1 nuclear accumulation and MKL1 target genes.** (**A**) The Rictor-KD HCCLM3 cells co-transfected with NLS-GFP and mCherry-MKL1 plasmids were starved under the low-serum conditions (1% FBS) for 6 hrs and then imaged by fluorescence microscopy (Scale bar: 10 µm). (**B**) The ratio of cells with MKL1 localization in the nuclei (N) or/and cytoplasm (**C**) among the total number of cells examined was monitored and analyzed by chi-square test. n≥50. (C) The overexpressing Rictor Hep3B cells co-transfected with NLS-GFP and mCherry-MKL1 plasmids were starved under the low-serum conditions (1% FBS) for 2 hrs and then imaged by fluorescence microscopy (Scale bar: 10 µm). (**D**) The ratio of MKL1 accumulation in the nuclei (N) or/and cytoplasm (C) to its total number was normalized to that in control cells and quantified by chi-square test. n≥50. (**E**) Rictor-specific siRNAs were transfected into HEK293T cells. Forty-eight hours after transfection of siRNA, PLG433 and beta-actin Renilla plasmids were cotransfected into the cells. Luciferase activity was measured using the dual luciferase reporter assay system. The fold change of luciferase activity normalized by Renilla is summarized from three independent experiments in duplicate. (**F**) HCCLM3 cells transfected with siRictors or siNC were subjected to Western blotting to detect the protein levels of Rictor and Arp3 and c-Fos. (**G**) The protein levels of Rictor, Arp3 and c-Fos were examined by Western blotting using the corresponding antibodies in Hep3B cells transfected with Rictor or control plasmids. (**H**) The mRNA levels of Rictor and Arp3 and c-Fos, were measured by real-time PCR in cells transfected with siRictors or siNC. Differences between the marked groups were quantified by two-tailed Student's t-test. (**I**) The mRNA levels of Arp3 and c-Fos were examined by real-time PCR experiments in Hep3B cells transfected plasmids expressing Rictor (Rictor OE) or control vector (Ctr).

MKL1 is a well-known coactivator of SRF (serum response factor), which mediates the transcription of multiple genes involved in diverse cellular processes, such as cell migration and tumor metastasis 38-40. It has been reported that the dynamics of actin polymerization induce MKL1 nuclear accumulation, subsequently activating MKL1-dependent genes 34, 41. To ask if Rictor regulates the subcellular localization of MKL1 in HCC cells, we cotransfected plasmids encoding NLS-GFP and mCherry-MKL1 into Rictor-KD HCCLM3 cells and fluorescence images were taken after cells were starved under the low-serum conditions (1% FBS). We found that MKL1 was predominantly localized in the nuclei of control cells, whereas MKL1 was markedly enriched in the cytoplasm in Rictor-KD cells. Quantification analysis revealed that the number of cells with MKL1 cytoplasmic localization induced by Rictor KD was approximately two-fold higher than that of controls (Figure 3A, B). Contrary to our observation, forced expression of Rictor in Hep3B cells promoted the MKL1 nuclear accumulation (Figure 3C, D). Collectively, these data indicate that Rictor is involved in the cellular distribution of MKL1 in HCC cells.

The notion that Rictor induces nuclear location of MKL1 prompts us to investigate whether Rictor activates SRF/MKL1-dependent gene transcription. To test this, we transfected luciferase reporter plasmids with a SRF/MKL1-binding promoter element into HEK293T cells with Rictor knockdown. The luciferase reporter activity was measured at 24 hrs post-transfection. The results demonstrated that downregulation of Rictor significantly inhibited luciferase activity compared with control (Figure 3E). Collectively, these data indicate that Rictor induces SRF/MKL1-dependent gene activation.

Next, we detected the expression levels of MKL1 target genes, including Arp3 and c-Fos. Western blotting assay and real-time PCR experiments demonstrated that Rictor knockdown significantly decreased the expression of Arp3 and c-Fos (Figure 3F, H), whereas Rictor overexpression resulted in increased levels of Arp3 and c-Fos (Figure 3G, I). To confirm our findings, we analyzed the gene expression correlation between Rictor and Arp3 or c-Fos in a clinical cohort from the TCGA database. The data showed that high expression levels of Rictor were positively associated with those of Arp3 and c-Fos in HCC tissues (Supplementary Figure S2A, B), consistent with the real-time PCR results. Taken together, our results suggest that Rictor plays an important role in actin polymerization in HCC cells.

### Rictor phosphorylates and interacts with ABLIM1 in HCC cells

Rictor, as a core component of mTORC2, mediates a series of protein activities through phosphorylation, such as AKT, SGK and PKC 26-30. To explore the molecular mechanism underlying the function of Rictor in cancer cell lines, we defined the Rictor-regulated phosphoproteome by quantitative mass spectrometry and analyzed the potential substrates of Rictor. We found that 55 phosphorylation sites from 42 proteins were down-regulated, and 23 phosphorylation sites from 21 proteins were up-regulated in the Rictor knockdown cells (Supplementary Table S2). Based on the involvement of Rictor in actin polymerization as described above, we focused on ABLIM1 (actin-binding LIM protein 1) as a critical candidate for further investigation.

**Figure 4:**
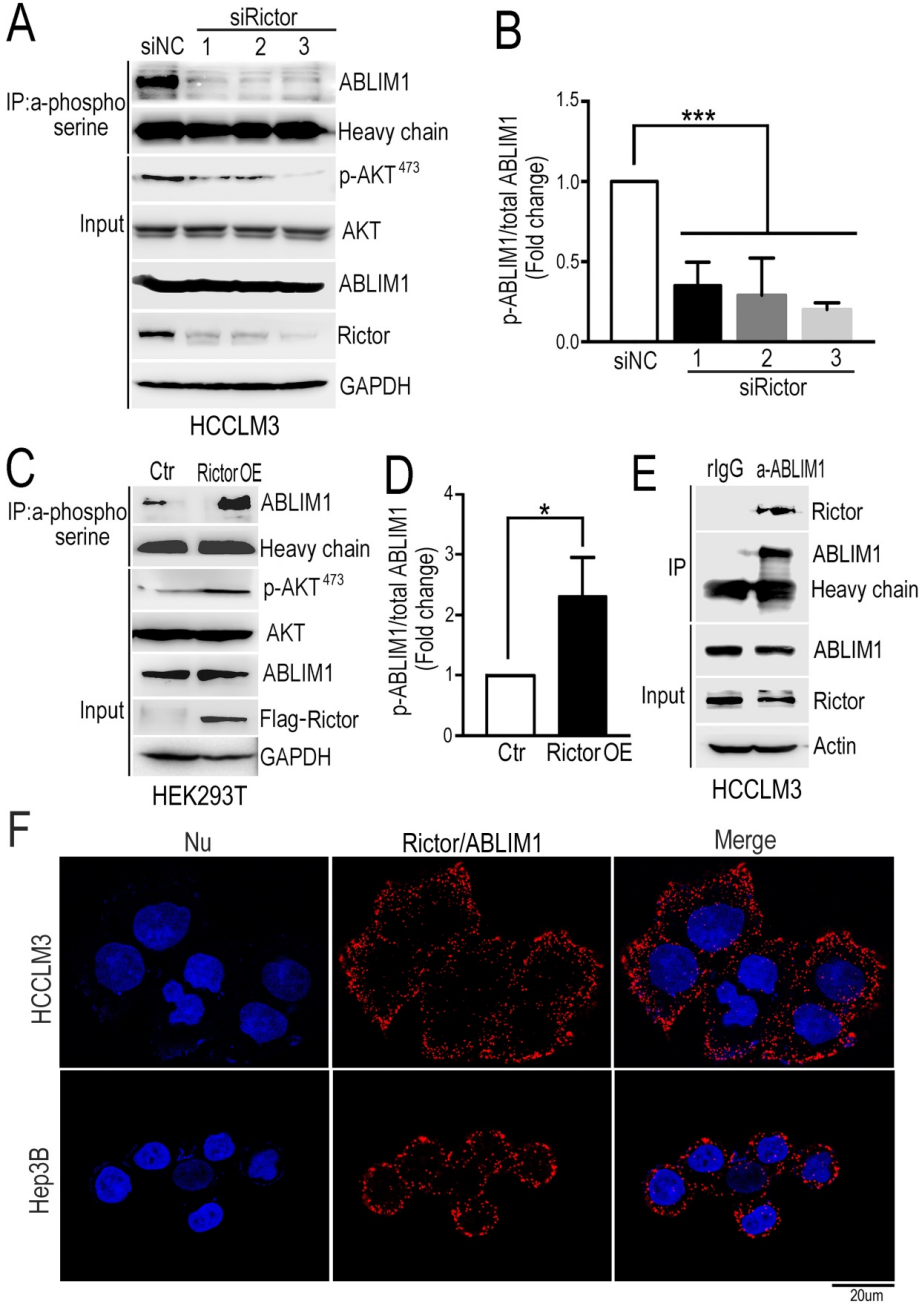
** Rictor phosphorylates and interacts with ABLIM1.** (**A**) HCCLM3 cells were transfected with three siRictors or siNC and cell lysates were prepared at 72 hrs post-transfection. The immunoprecipitation (IP) assay was performed with phosphoserine antibodies coupled with protein A dynabeads, followed by immunoblotting using anti-ABLIM1 antibodies. Heavy chain was utilized as an internal control of IP. Proteins extracted from the total cell lysates were subjected to Western blotting to detect the expression of corresponding proteins indicated in Input panels. GAPDH was used as a loading control. (**B**) The ratio of phosphorylated ABLIM1 to total ABLIM1 protein levels was quantified by one-way ANOVA test and shown as the means±SEM of three independent experiments. ***: *p*<0.0001. (**C**) Plasmids expressing Rictor tagged with a flag epitope (Rictor OE) or flag control vector (Ctr) were transfected into HEK293T cells. Immunoprecipitation was carried out with phosphoserine antibodies to detect the phosphorylation of ABLIM1. Immunoprecipitated proteins were examined by Western blotting with anti-ABLIM1 antibodies. Input shows the expression of corresponding proteins in the total cell lysates. (**D**) Phosphorylated levels of ABLIM1 from three independent experiments were examined by Student's *t*-test. *: *p*<0.05. (**E**) Co-immunoprecipitation assay was carried out to detect the interaction of endogenous Rictor with ABLIM1 in HCCLM3 cells. Monoclonal anti-ABLIM1 antibodies were utilized for immunoprecipitation and immunoprecipitated proteins were detected by Western blotting with anti-Rictor antibodies. (**F**) HCCLM3 and Hep3B cells were respectively fixed with 4% PFA, followed by permeabilization with methonal. After incubation with mouse anti- Rictor and rabbit anti-ABLIM1 antibodies, cells were stained with two secondary antibodies linked to PLA probes (one PLUS and one MINUS). The probes hybridized to circular DNA were amplified with fluorescently-labeled oligonucleotide. The spots of proximity were visualized by fluorescence microscopy (Scale bar: 10 µm).

To validate the role of Rictor in regulating ABLIM1 phosphorylation in HCC cells, we knocked down endogenous Rictor by transfecting HCCLM3 cells with three siRictors or siNC, respectively. First, we examined the phosphorylation of AKT at Ser 473 residue in HCC cells with Rictor KD, because it is a well-known substrate for mTORC2 and its activation is a hallmark of tumor progression, including for HCC 18, 42. Consistent with the studies in other cancers 28-30, Western blotting demonstrated that pAKT was dramatically repressed by Rictor downregulation in HCC cells (Figure 4A, B).

Analysis from our phosphoproteomics data showed that phosphorylation of ABLIM1 at serine residues was decreased when Rictor was downregulated in cancer cell lines (Supplementary Table S2). Then, immunoprecipitation was carried out in Rictor-KD cells with phosphoserine antibodies coupled with dynabeads, followed by immunoblotting with anti-ABLIM1 antibodies. Consistently, the phosphorylated levels of ABLIM1 at serine sites were dramatically reduced in Rictor KD cells compared to the control, indicating that Rictor regulates ABLM1 phosphorylation in HCC cells. To confirm this finding, we examined the effect of Rictor on ABLIM1 phosphorylation in HEK293T cells overexpressing Rictor. As expected, overexpression of Rictor significantly increased the phosphorylation levels of ABLIM1 (Figure 4C, D), contrary to the observation in Rictor-KD cells. Overall, we identified ABLIM1 as a novel phosphorylation target of Rictor in HCC cells.

Next, we asked if Rictor regulates ABLIM1 phosphorylation by carrying out coimmunoprecipitation experiment to test the interaction between endogenous Rictor and ABLIM1 in HCC cells. After immunoprecipitation using the control IgG or anti-ABLIM1 antibodies, the immunoprecipitated proteins were detected by Western blotting using anti-Rictor antibodies. The results showed that anti-ABLIM1 antibodies coimmunoprecipitated Rictor in HCCLM3 cells, whereas control IgG did not, suggesting that ABLIM1 is associated with Rictor in HCC cells (Figure 4E).

Next, we determined the interaction of Rictor and ABLIM1 *in situ* using proximity ligation assay (PLA). HCCLM3 and Hep3B cells were respectively fixed with PFA, followed by incubation with the mixture of anti-Rictor and anti-ABLIM1 antibodies. Intramolecular interaction was detected and the spots of proximity were visualized by fluorescence microscopy. As shown in the Figure 4F, dots were distributed around the nuclei, supporting that endogenous Rictor-ABLIM1 interaction in HCC cells.

To further strengthen our findings, we measured the colocalization of Rictor and ABLIM1 in HCCLM3 cells. Immunofluorescence was performed with anti-Rictor, anti-ABLIM1, or negative control IgG in different combinations, and representative images were captured by confocal microscopy. As described previously 32-33, ABLIM1 was distributed throughout the cells, whereas Rictor was mainly expressed in the cytoplasm. Overlapping images demonstrated the partial colocalization of Rictor and ABLIM1 in the cytoplasm of HCC cells (Supplementary Figure S2C). Furthermore, neither of two proteins colocalized with the corresponding negative control, indicating that Rictor specifically interacts with ABLIM1 in HCC cells.

### ABLIM1 knockout inhibits HCC cell migration

**Figure 5:**
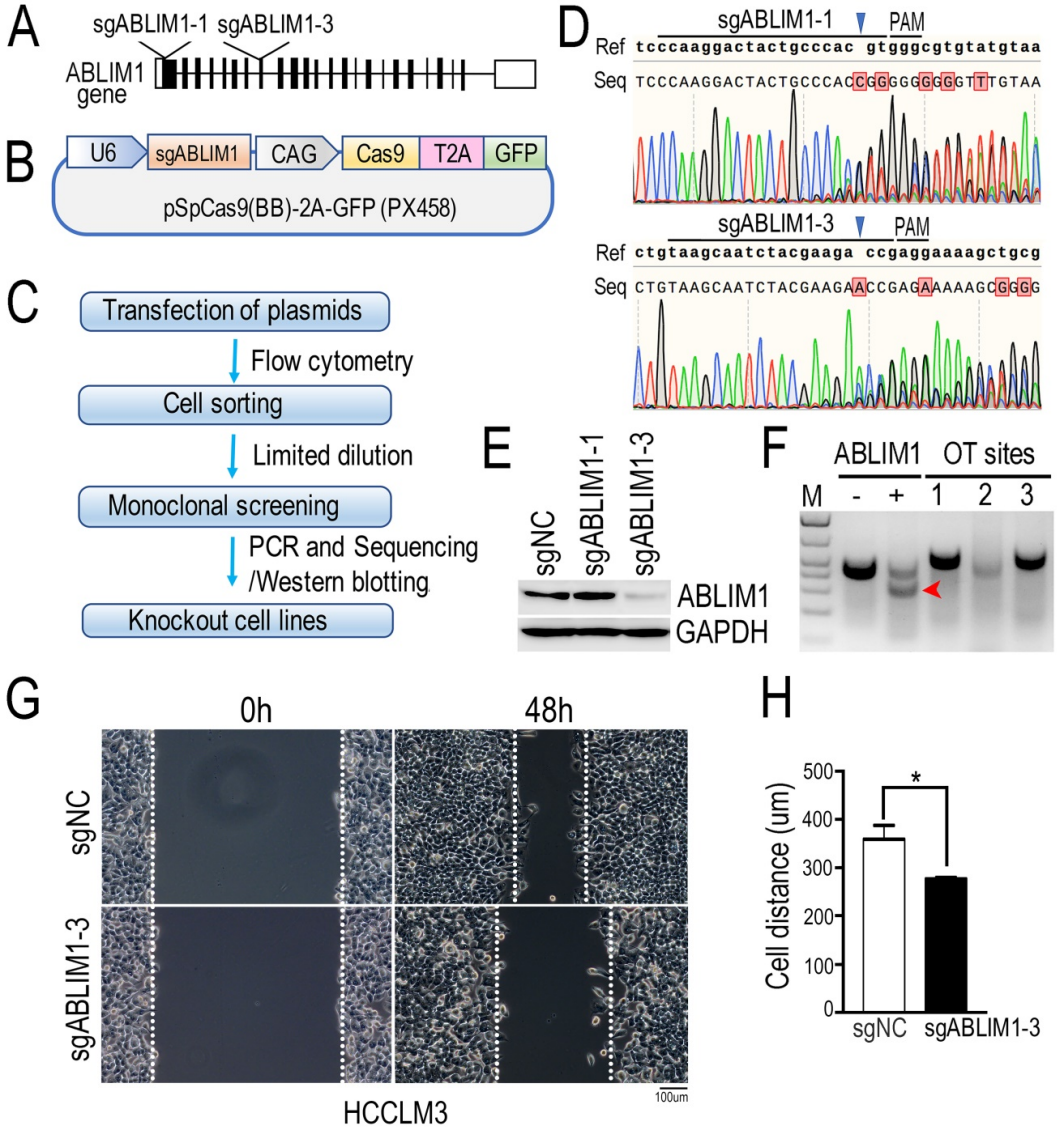
** ABLIM1 knockout suppresses the migration of HCC cells.** (**A**) Schematic depicting the two sgRNAs targeting the exons of ABLIM1 gene for gene editing. (**B**) An illustration demonstrating that annealing sgRNAs were inserted into the pSpCas9(BB)-2A-GFP (PX458) vector. (**C**) The workflow illustrates the screening of a single ABLIM1 KO cell line by FACS based on GFP fluorescence. (**D**) The DNA sequencing data show the multiple peaks after the corresponding PAM (protospacer adjacent motif). (**E**) Protein levels of ABLIM1 were examined by Western blotting using anti-ABLIM1 antibodies in the selected ABLIM1-KO or control HCCLM3 cells. GAPDH was used as an internal control. (**F**) The T7E1 nuclease was used to assess the off-target (OT) effect of sgRNA-3 targeting ABLIM1 (sgABLIM1-3) genome loci. -, negative control; +, positive control; OT sites 1,2,3 represented the top-ranking off-target sites of sgABLIM1-3. The red arrow indicates the cleaved band by the T7E1 nuclease in cells, where sgABLIM1-3 induced genomic editing. (**G**) After ABLIM1-KO cells induced by sgABLIM1-3 were plated onto fibronectin-coated coverslips and incubated with media in the presence of mitomycin C (5 µM), wound scratches were formed so that cells migrated toward the center of the wound area from the edge marked by the dotted lines. Representative images taken at 0 hr and 48 hrs are shown. (Scale bar: 100 µm). (**H**) Quantification of cell migration distance during the 48-hr period. The data are presented as the means±SEM from three independent experiments. *: p<0.05 by Student's *t*-test.

To investigate the molecular mechanism underlying ABLIM1 function in HCC cells, we generated ABLIM1 knockout (KO) HCCLM3 cells by CRISPR-Cas9 technology. As shown in the Figure 5A and B, we designed two specific sgRNAs targeting the exons of ABLIM1 gene. Then, we constructed CRISPR-sgABLIM1 plasmids with the pSpCas(BB)-2A-GFP (PX458) vector, which expresses the Cas9 endonuclease fused to GFP through the T2A peptide. To validate the effect of sgRNAs in editing the ABLIM1 genomic loci, HCCLM3 cells transiently transfected with PX458-sgABLIM1 plasmids were sorted by flow cytometry based on GFP fluorescence. According to the workflow indicated in Figure 5C, monoclonal cells were obtained by limited dilution, followed by amplification. Genomic DNA was generated by PCR with primer pairs located outside the two sgRNAs and sequenced to analyze the ability of sgRNAs to induce ABLIM1 genomic editing. As shown in Figure 5D, sgABLIM1-1 guided Cas9 to cleave the ABLIM1 gene at three bases upstream of the proto-spacer adjacent motif (PAM), leading to the insertion, whereas sgABLIM1-3 induced to deletion of the genomic DNA of ABLIM1 at the predicted sites of PAM. Furthermore, we examined the protein levels of ABLIM1 by Western blotting using anti-ABLIM1 antibodies. We found that ABLIM1 protein was dramatically abolished in sgABLIM1-3-induced cells, but not in control cells or sgABLIM1-1-induced cells (Figure 5E), consistent with the sequencing data. Taken together, specific sgABLIM1-3 induced the deletions of ABLIM1 gene loci, resulting in inhibition of ABLIM1 expression. To characterize the potential off-target effects of sgABLIM1-3 in cells, we identified three top-ranking sgABLIM1-3 off-target genomic sites using T7E1 assay. The data revealed that no T7E1 nuclease cleavage was detected at the off-target sites (Figure 5F). Overall, we generated the stable ABLIM1-KO HCCLM3 cells using the CRISPR-Cas9 system. The ABLIM-KO HCCLM3 cells induced by sgABLIM1-3 were used in the subsequent experiments. To understand the function of ABLIM1 signaling in HCC cells, wound-healing experiments were performed with ABLIM-KO cells to determine the effect of ABLIM1 on cell migration. ABLIM-KO cells exhibited lower migration speed than the control cells, indicating that ABLIM1 promotes HCC cell migration (Figure 5G, H).

### ABLIM1 regulates actin polymerization

**Figure 6:**
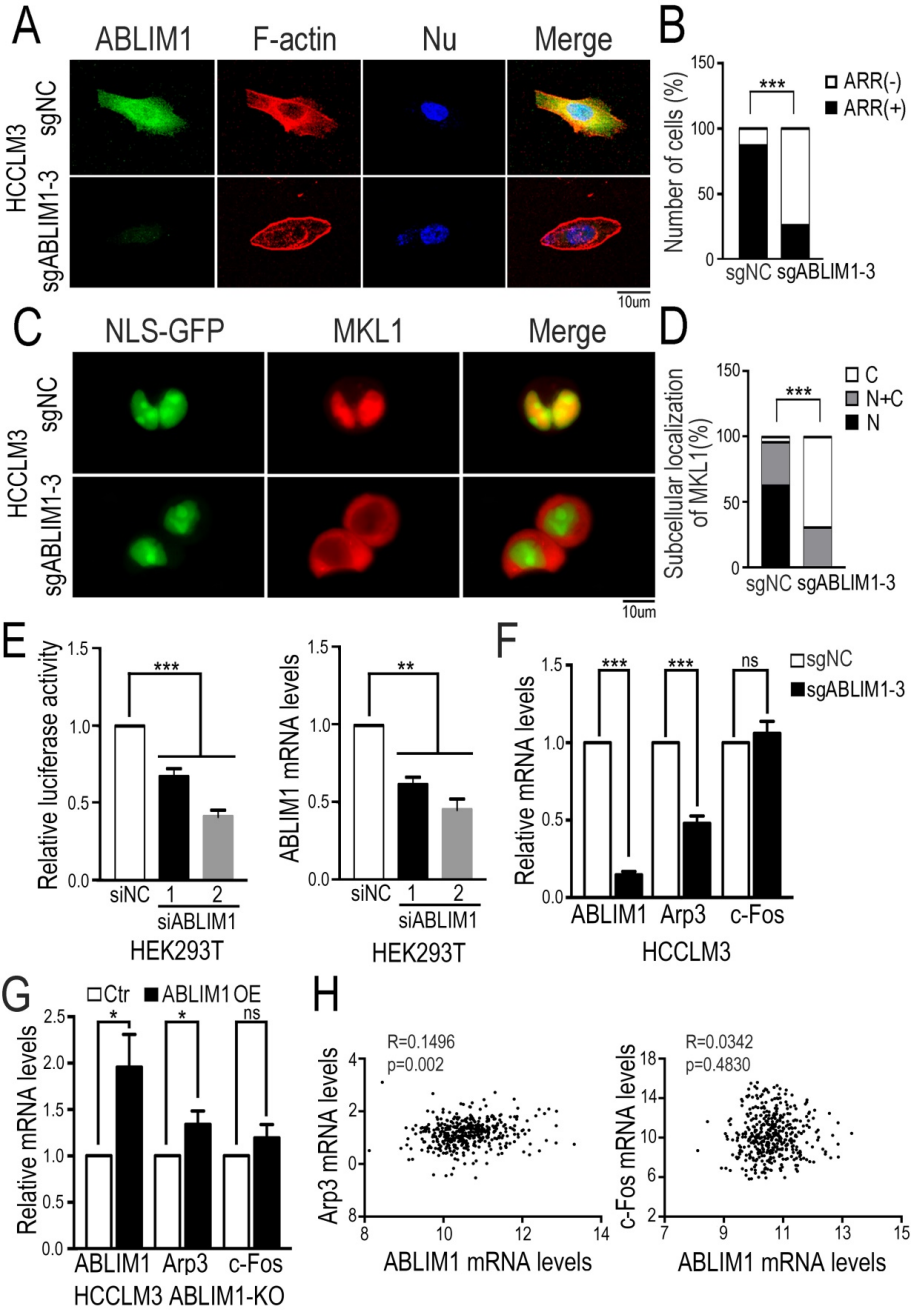
** ABLIM1 knockout impairs actin polymerization.** (**A**) ABLIM1-KO or control HCCLM3 cells were fixed and stained with primary antibodies against ABLIM1 proteins, and immunocomplexes were detected with Alexa488-conjugated secondary antibodies and Alexa594-conjugated phalloidin respectively. Nuclei were stained with DAPI. Fluorescence images were photographed by confocal microscopy. (Scale bar: 10 µm). (**B**) Cells with actin-rich regions were quantified by chi-square test from at least 50 cells per group. (**C**) The plasmids encoding GPF fused to the NLS epitope (GFP-NLS) and MKL1 tagged with mCherry were co-transfected into ABLIM1-KO or control HCCLM3 cells, followed by starvation for 2 hrs with 1%FBS media. Shown are representative live images from three independent experiments (Scale bar: 10 µm). (**D**) Subcellular localization of MKL1-mCherry in each group was quantified by chi-square test. n≥50. (**E**) HEK293T cells transfected with two siRNAs against ABLIM1 were transfected with PGL433 and beta-actin Renilla plasmids. Luciferase activity was measured at 24 hrs post-transfection. The efficiency of ABLIM1 knockdown was examined by real-time PCR assay. The results shown are summarized from three independent experiments in duplicate. P-values were determined by Student's *t*-test. ***: *p*<0.0001; **: *p*<0.001. (**F**) Real-time PCR was performed to assess expression of Arp3 and c-Fos in ABLIM1-KO or control HCCLM3 cells. P-values were determined by Student's *t*-test. ***: *p*<0.0001. (**G**) The mRNA levels of Arp3 and c-Fos were examined by real-time PCR experiments in ABLIM1-KO cells transfected with plasmids encoding ABLIM1 (ABLIM1 OE) or control vector (Ctr). (**H**) The gene expression correlation of ABLIM1 and Arp3 or c-Fos in HCC tissues was analyzed from the TCGA dataset. Relative mRNA levels of ABLIM1 were respectively plotted against those of Arp3 or c-Fos. R represents a Pearson coefficient and the *p*-value is shown.

To clarify the role of ABLIM1 in HCC cells, we investigated its impact on actin skeleton regulation. Immunostaining of F-actin with fluorescently labeled phalloidin revealed more enrichment of F-actin structure in control cells, such as membrane ruffles and stress fibers, compared with ABLIM1-KO cells (Figure 6A, B), suggesting that ABLIM1 promotes F-actin formation in HCC cells, consistent with previous reports 32-33. Next, we examined whether ABLIM1 is required for nuclear localization of MKL1 in HCC cells. Fluorescence microscopy was performed in ABLIM1-KO or control cells transfected with NLS-GFP and mCherry-MKL1 plasmids. In control cells treated with media containing 1% FBS, the majority of MKL1 protein signal was detected in the nuclei, whereas ABLIM1 KO contributed to the redistribution of MKL1 from the nuclear region to the cytoplasm (Figure 6C, D).

We next asked whether ABLIM1 affected MKL1-targeting gene transcription. Luciferase reporter assay demonstrated that ABLIM1 knockdown decreased SRF/MKL1-dependent luciferase reporter activity (Figure 6E). Subsequently, real-time PCR experiments showed that Arp3 expression was markedly reduced in ABLIM1-KO cells, whereas the expression levels of c-Fos were not significantly altered (Figure 6F). Moreover, we transfected plasmids expressing ABLMI1 proteins into ABLIM1-KO cells and real-time PCR experiments showed that ectopic ABLIM1 expression rescued the inhibitory effect of ABLIM1 KO on Arp3 activation, but not c-Fos (Figure 6G). The data from TCGA dataset showed that expression of ABLIM1 in HCC tissues positively correlated with that of Arp3, not c-Fos (Figure 6H), supporting our notion that ABLIM1 specially activates MKL1 target gene, Arp3. Collectively, these data suggest that ABLIM1 induces increased nuclear MKL1 localization, leading to activation of Arp3, a key factor for actin polymerization 43-44.

### Both Ser 214 and Ser 431 residues are required for ABLIM1 localization to the cell membrane and ABLIM1-mediated MKL1 nuclear accumulation

To characterize whether changes in ABLIM1 phosphorylation are required for ABLIM1 to mediate actin polymerization, we generated a series of ABLIM1 mutations. In our phosphoproteomics data, we found that phosphorylation of ABLIM1 at Ser214 and Ser431 was regulated by Rictor (Supplementary Table S2). To investigate the function of both serine residues on ABLIM1 downstream signaling pathway, we constructed sequential mutants of ABLIM1, in which the corresponding serine residues were substituted with phosphomimicking asparticacid (D) or non-phosphorylatable alanine (A), generating constitutively active and dominant negative forms of ABLIM1, respectively.

**Figure 7:**
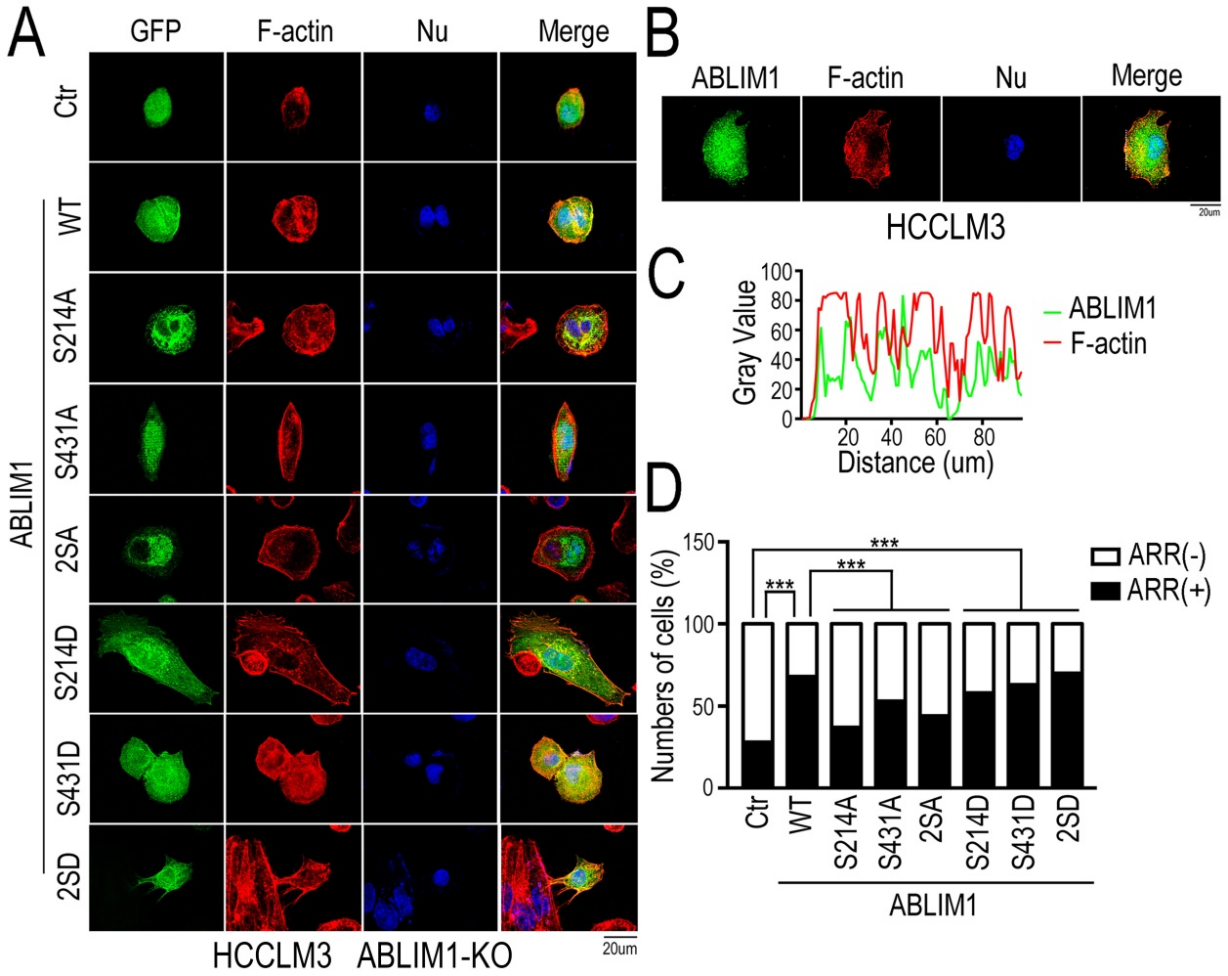
** Both Ser 214 and Ser 431 residues of ABLIM1 are required for the colocalization of ABLIM1 and F-actin at the cell membrane.** (**A**) The plasmids encoding GFP-ABLIM1 wild type (WT) and a series of indicated GFP-ABLIM1 mutants were constructed and each of these plasmids was transfected into ABLIM1-KO HCCLM3 cells. Phalloidin staining was performed to visualize the F-actin network, and DAPI staining marked the cell nucleus. The cellular distribution of ABLIM1 was observed by fluorescence microscopy. The colocalization of ABLIM1 mutants and F-actin at the cell membrane was indicated. Scale bar: 20 µm. (**B**) Immunofluorescence staining of ABLIM1 (green) and F-actin (red) was performed with anti-ABLIM1 specific antibodies and fluorescently labelled phalloidin in HCCLM3 cells. DAPI was used for nuclear staining; scale bar 20 µm. (**C**) The line-scan analysis of ABLIM1 and F-actin along the white dotted line is depicted in the merged images using ImageJ software. (**D**) The ratio of cells with high actin-rich regions among the total number of cells transfected with GFP-ABLIM1 mutants was quantified by chi-square test. At least 50 cells were counted. ***: *p*<0.0001.

We evaluated whether the ABLIM1 mutants could alter the membrane colocalization of ABLIM1 with F-actin, a critical process for subsequent actin polymerization. Fluorescence microscopy was carried out in ABLIM1-KO cells transfected with a series of plasmids encoding ABLIM1 mutants fused to GFP, followed by phalloidin immunostaining to visualize F-actin (Figure 7A). The results uncovered that wild type (WT) ABLIM1 partially colocalized with F-actin at the cell membrane, consistent with our data from colocalization of endogenous ABLIM1 and F-actin at the cell membrane (Figure 7B, C) and previous studies 18,19,21. The dominant negative forms of ABLIM1 (S214A, S431A and S214A/S431A) primarily distributed at the pre-nuclear region and their colocalization with F-actin at the cell membrane was rarely observed. Moreover, either or two active ABLIM1 mutants (S214D, S431D or S214D/S431D) could constitutively colocalize with F-actin at cell membrane, similar to WT. More importantly, the incorporation of activating ABLIM1 mutants in F-actin induced the formation of cell protrusions as detected by phalloidin staining in ABLIM1-KO cells, whereas dominant negative forms blocked ABLIM1-mediated actin-rich accumulation in these cells (Figure 7D). Overall, inhibition of ABLIM1 phosphorylation at Ser214 and Ser431 residues reduced the ABLIM1 localization at the cell membrane, indicating that they may be required for the activation of ABLIM1 signaling pathway.

**Figure 8:**
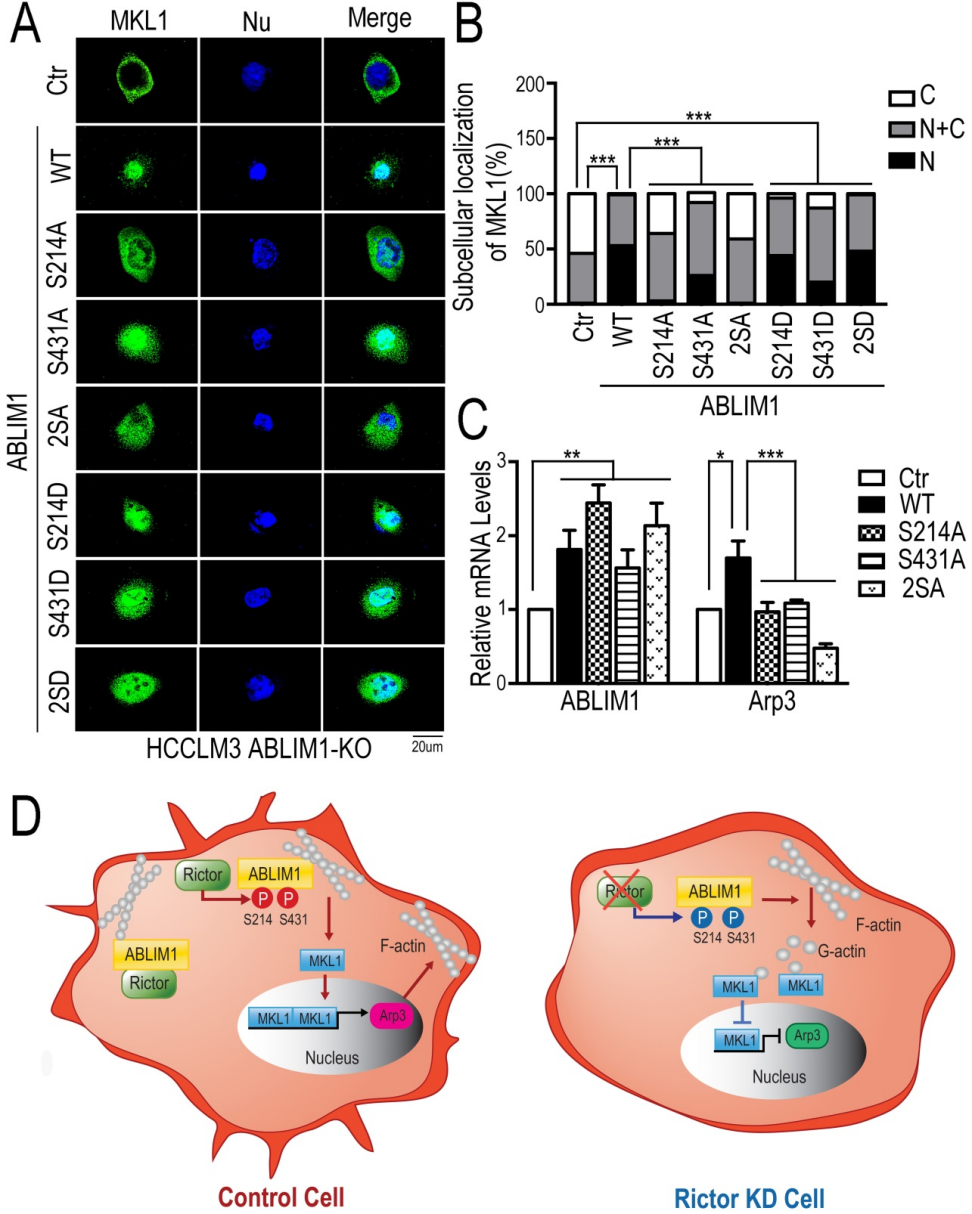
** Both Ser 214 and Ser 431 residues of ABLIM1 are involved in the nuclear accumulation of MKL1 and activation of Arp3.** (**A**) HCCLM3 cells transfected with a series of plasmids expressing ABLIM1 mutants were stained with anti-MKL1 antibodies, followed by incubation with Alexa488-conjugated secondary antibodies. Representative images were photographed using confocal fluorescence microscopy. (Scale bar: 20 µm). (**B**) The ratio of MKL1 immunofluorescence intensity in nucleus (N), nucleus and cytoplasm (N+C), or cytoplasm (C) among total cells was quantified by chi-square test. At least 50 cells were counted. ***: *p*<0.0001. (**C**) Real-time quantitative PCR was carried out to evaluate the relative mRNA levels of ABLIM1 and Arp3 in ABLIM1-KO cells transfected with indicated dominant negative mutants of ABLIM1. Differences between the different groups were quantified by one-way ANOVA test. (**D**) Schematic diagram illustrating a working model for ABLIM1 phosphorylation induced by Rictor involving in actin polymerization. In control cells, Rictor interacts with ABLIM1 and phosphorylates ABLIM1, thereby leading to actin polymerization and accumulation of MKL1 in the nucleus, where it promotes expression of Arp3. In HCC cells with Rictor knockdown, phosphorylated levels of ABLIM1 are dramatically decreased, which results in reduction of F-actin formation. Free G-actin can bind to MKL1 in the cytosol, resulting in its cytoplasmic retention. As a consequence, MKL1 fails to be translocated into the nucleus and activate Arp3 expression.

To further validate our findings, we performed the immunostaining assay to detect the distribution of MKL1 in ABLIM1-KO cells transfected with a series of ABLIM1 mutants. We found that ABLIM1-KO cells stimulated with control vector displayed the predominant enrichment of MKL1 in the cytoplasm, whereas ABLIM1 WT transfection facilitated the nuclear accumulation of MKL1. Similar to the wild type, active mutants (S214D, S431D or S214D/S431D) retained more MKL1 signals in the nucleus. However, the expression of the dominant-negative constructs (S214A, S431A and S214A/S431A) failed to significantly import MKL1 into the nucleus, suggesting that Ser 214 and Ser 431 residues of ABLIM1 are required for nuclear accumulation of MKL1(Figure 8A, B). In line with a critical role for the two serine residues in mediating MKL1 localization, these non-phosphorylatable mutants (S214A, S431A and S214A/S431A) could not further stimulate the activation of Arp3 (Figure 8C). In summary, the expression of dominant negative ABLIM1 decreased its cell membrane localization and resulted in cytoplasmic retention of MKL1, thereby impairing the subsequent activation of MKL1 target gene. These data reveal that regulation of ABLIM1 on actin polymerization requires the phosphorylation of ABLIM1 at Ser 214 and Ser 431 residues in HCC cells.

### ABLIM1 is frequently up-regulated in HCC tissues and correlates with poor prognosis for patients

To evaluate the function of ABLIM1 in liver cancer pathogenesis, ABLIM1 expression was analyzed in HCC using the Oncomine database. ABLIM1 expression levels were significantly higher in HCC samples than those in non-cancerous liver tissues (Supplementary Figure S3A). The data from the cBioPortal database demonstrated that the frequency of genetic alterations of *ABLIM1* in liver cancer accounted for approximately 0.4~0.8%, including mutations (Supplementary Figure S3B). Notably, three somatic mutations resided inside the domains of ABLIM1 (Supplementary Figure S3C). In the GEPIA database in which survival data are available, the correlation between ABLIM1 expression and patient survival in HCC was further interrogated. Higher ABLIM1 expression predicted a shorter overall survival (OS) time for HCC patients (Supplementary Figure S3D). Overall, ABLIM1 may serve as a tumor-promoting gene and has a potential prognostic prediction value in HCC.

## Discussion

Rictor, a key subunit of mTORC2 which has been implicated in various cellular processes, is up-regulated in several cancers, such as breast cancer and pancreatic cancer 19, 30. However, the role of Rictor in HCC remains largely unknown. In this study, our analysis demonstrated that Rictor is dramatically elevated in HCC samples, consistent with previous studies of other cancers 19, 30. Genetic changes that alter activity, abundance and cellular distribution of proteins were observed in wide variety of malignancies. In liver cancer, alteration frequency of the *RICTOR* gene accounts for approximately 4% of patient cases, including amplification, shows that the *RICTOR* gene may be amplified or overexpressed in HCC tissues. Additionally, HCC patients with higher Rictor expression in tumor tissues exhibit shorter OS and DFS time. Our analysis indicates that Rictor may act as a potential diagnostic and prognostic biomarker for HCC patients.

We found that knockdown of Rictor inhibited the migration of HCC cells, which is in line with previous studies of other cancers 27-30. Moreover, we found that Rictor knockdown abolished actin polymerization, subsequently reducing nuclear location of transcriptional co-activator MKL1. The reduction of MKL1 in nuclei impaired the induction of MKL1-dependent genes, Arp3 and c-Fos. Taken together, our results support the evidence that Rictor, a novel regulator of the MKL1 signaling pathway, plays a critical role in meditating the migration and actin polymerization of HCC cells.

ABLIM1 has been reported to interact with F-actin to promote actin polymerization 32-34. However, the molecular mechanism underlying this regulation by phosphorylation in HCC cells remained unclear. Our phosphoproteome analysis identified ABLIM1 as a previously unknown substrate of Rictor. Specifically, loss of Rictor resulted in the decreased serine phosphorylation of ABLIM1; in turn, overexpression of Rictor promotes phosphorylation of ABLIM1. Moreover, we revealed that endogenous Rictor interacted with ABLIM1, suggesting that Rictor induces the phosphorylation of ABLIM1 through interacting with it in HCC cells. Therefore, we provide the first evidence demonstrating that ABLIM1 serves as a phosphorylation substrate of Rictor in HCC cells.

Mechanistically, the ABLIM1-KO suppressed actin polymerization and nuclear localization of MKL1, similar to the observation in Rictor-KD. Our phosphoproteome analysis predicted that both serine 214 and serine 431 residues of ABLIM1 were the potential phosphorylation sites targeted by Rictor. Sequential mutagenesis of the serine residues revealed that Ser 214 and Ser 431 residues were required for colocalization of ABLIM1 and F-actin at plasma membrane. Furthermore, dominant negative mutations impaired the ABLIM1-induced F-actin formation and nuclear distribution of MKL1 and activation of Arp3, which is a key factor for actin nucleation 43-44. Collectively, our data indicate that phosphorylated Ser 214 and Ser 431 residues of ABLIM1 induced by Rictor may serve as docking sites to allow ABLIM1 to recruit into the plasma membrane to promote actin polymerization. In the future, inhibition by ABLIM1 phosphorylation on the two serine residues could be used as a novel therapeutic strategy for HCC.

Overall, as illustrated in our working model depicted in Figure 8D, Rictor acts to promote cell migration and cell protrusion formation. Rictor knockdown renders MKL1 to accumulate in the cytoplasm, leading to the inhibition of Arp3 expression. Rictor interacts with ABLIM1 and phosphorylates ABLIM1 at Ser 214 and Ser 431 residues in HCC cells. Moreover, both Ser 214 and Ser 431 residues are required for the ABLIM1 colocalization with F-actin at the cell membrane and the ABLIM1-induced MKL1 nuclear distribution and activation of Arp3. These data, to our knowledge, uncover a previously unrecognized role of ABLIM1 phosphorylation induced by Rictor in HCC.

Of note, our analysis has shown that increased ABLIM1 expression in HCC tissue samples predicts the shorter overall survival time, suggesting that ABLIM1 may be a potential prognosis biomarker for HCC. We hypothesize that co-targeting both Rictor and ABLIM1 strategy may provide a promising diagnostic and therapeutic benefit for HCC patients.

## Conclusions

The tissue-array evaluation and/or bioinformatics analysis demonstrated that the elevated expression of Rictor and ABLIM1 in HCC predicts poorer prognosis for HCC patients, implicating them as two promising diagnostic and prognostic indicators for HCC patients. Our functional experiments showed that Rictor regulates cell migration and actin polymerization in HCC cells. Rictor knockdown suppressed MKL1 nuclear accumulation, resulting in reduced Arp3 expression. Rictor interacts with ABLIM1 to promote phosphorylation of ABLIM1 at Ser 214 and Ser 431 residues in HCC cells. Furthermore, both Ser 214 and Ser 431 residues are required for ABLIM1 colocalization with F-actin at the cell membrane and the ABLIM1-induced MKL1 nuclear distribution and activation of Arp3. Overall, the findings suggest that co-targeting Rictor and ABLIM1 may have translational potential for further HCC management.

## Supplementary Material

Supplementary figures and tables.

## Abbreviations

ABLIM1: actin-binding LIM1
ARR: actin-rich region
CCK-8: Cell counting kit-8
coIP: coimmunoprecipitation
CRISPR/Cas9: clustered regularly interspaced short palindromic repeats/CRISPR-associated protein 9
DFS: disease free survival
DMEM: Dulbecco's modified Eagle's medium
HCC: hepatocellular carcinoma
IF: immunofluorescence
KD: knockdown
KO: knockout
OS: overall survival
PAM: protospacer adjacent motif
Rictor: rapamycin insensitive companion of mTOR
sgRNA: single guide RNA
TCGA: The cancer genome atlas
WB: western blotting

## References

[1] Villanueva A (2019). Hepatocellular carcinoma. N Engl J Med.

[2] Siegel RL, Miller KD, Jemal A (2018). Cancer statistics, 2018. CA Cancer J Clin.

[3] Forner A, Llovet JM, Bruix J (2012). Hepatocellular carcinoma. Lancet.

[4] Kulik L, El-Serag HB (2019). Epidemiology and Management of Hepatocellular Carcinoma. Gastroenterology.

[5] Song D, Ye Q, Carpenito C, Poussin M, Wang L, Ji C (2011). *In vivo* persistence, tumor localization, and antitumor activity of CAR-engineered T cells is enhanced by costimulatory signaling through CD137 (4-1BB). Cancer Res.

[6] Greten TF, Lai CW, Li G, Staveley-O'Carroll KF (2019). Targeted and immune-based therapies for hepatocellular carcinoma. Gastroenterology.

[7] Llovet JM, Montal R, Sia D, Finn RS (2018). Molecular therapies and precision medicine for hepatocellular carcinoma. Nat Rev Clin Oncol.

[8] Reig M, da Fonseca LG, Faivre S (2018). New trials and results in systemic treatment of HCC. J Hepatol.

[9] Qin J, Zhou Z, Chen W, Wang C, Zhang H, Ge G (2015). BAP1 promotes breast cancer cell proliferation and metastasis by deubiquitinating KLF5. Nat Commun.

[10] Saxton RA, Sabatini DM (2017). mTOR signaling in growth, metabolism, and disease. Cell.

[11] Mossmann D, Park S, Hall MN (2018). mTOR signalling and cellular metabolism are mutual determinants in cancer. Nature Rev Cancer.

[12] O'Donnell JS, Massi D, Teng MWL, Mandala M (2018). PI3K-AKT-mTOR inhibition in cancer immunotherapy, redux. Semin Cancer Biol.

[13] Jiang D, Wei S, Chen F, Zhang Y, Li J (2017). TET3-mediated DNA oxidation promotes ATR-dependent DNA damage response. EMBO Rep.

[14] Gulhati P, Bowen KA, Liu J, Stevens PD, Rychahou PG, Chen M (2011). mTORC1 and mTORC2 regulate EMT, motility, and metastasis of colorectal cancer via RhoA and Rac1 signaling pathways. Cancer Res.

[15] Yin Y, Hua H, Li M, Liu S, Kong Q, Shao T (2016). mTORC2 promotes type I insulin-like growth factor receptor and insulin receptor activation through the tyrosine kinase activity of mTOR. Cell Res.

[16] Chantaravisoot N, Wongkongkathep P, Loo JA, Mischel PS, Tamanoi F (2015). Significance of filamin A in mTORC2 function in glioblastoma. Mol Cancer.

[17] Xu J, Pham CG, Albanese SK, Dong Y, Oyama T, Lee C-H (2016). Mechanistically distinct cancer-associated mTOR activation clusters predict sensitivity to rapamycin. J Clin Invest.

[18] Grabiner BC, Nardi V, Birsoy K, Possemato R, Shen K, Sinha S (2014). A diverse array of cancer-associated mTOR mutations are hyperactivating and can predict rapamycin sensitivity. Cancer Discov.

[19] Driscoll DR, Karim SA, Sano M, Gay DM, Jacob W, Yu J (2016). mTORC2 signaling drives the development and progression of pancreatic cancer. Cancer Res.

[20] Song X, Liu X, Wang H, Wang J, Qiao Y, Cigliano A (2019). Combined CDK4/6 and pan-mTOR inhibition is synergistic against intrahepatic cholangiocarcinoma. Clinical Cancer Res.

[21] Zhou Q, Lui VWY, Yeo W (2011). Targeting the PI3K/Akt/mTOR pathway in hepatocellular carcinoma. Future Oncol.

[22] Dibble CC, Asara JM, Manning BD (2009). Characterization of Rictor phosphorylation sites reveals direct regulation of mTOR complex 2 by S6K1. Mol Cell Biol.

[23] Zhou P, Zhang N, Nussinov R, Ma B (2015). Defining the domain arrangement of the mammalian target of rapamycin complex component Rictor protein. J Comput Biol.

[24] Kaibori M, Shikata N, Sakaguchi T, Ishizaki M, Matsui K, Iida H (2015). Influence of Rictor and Raptor expression of mTOR signaling on long-Term outcomes of patients with hepatocellular carcinoma. Dig Dis Sci.

[25] Jacinto E, Loewith R, Schmidt A, Lin S, Rüegg MA, Hall A (2004). Mammalian TOR complex 2 controls the actin cytoskeleton and is rapamycin insensitive. Nat Cell Biol.

[26] Rispal D, Eltschinger S, Stahl M, Vaga S, Bodenmiller B, Abraham Y (2015). Target of rapamycin complex 2 regulates actin polarization and endocytosis via multiple pathways. J Biol Chem.

[27] Huang L, Zhang Y, Xu C, Gu X, Niu L, Wang J (2017). Rictor positively regulates B cell receptor signaling by modulating actin reorganization via ezrin. PLoS Biol.

[28] Sarbassov DD, Guertin DA, Ali SM, Sabatini DM (2005). Phosphorylation and regulation of Akt/PKB by the rictor-mTOR complex. Science.

[29] McDonald PC, Oloumi A, Mills J, Dobreva I, Maidan M, Gray V (2008). Rictor and integrin-linked kinase interact and regulate Akt phosphorylation and cancer cell survival. Cancer Res.

[30] Zhang F, Zhang X, Li M, Chen P, Zhang B, Guo H (2010). mTOR complex component Rictor interacts with PKCzeta and regulates cancer cell metastasis. Cancer Res.

[31] Jebali A, Dumaz N (2018). The role of RICTOR downstream of receptor tyrosine kinase in cancers. Mol Cancer.

[32] Roof DJ, Hayes A, Adamian M, Chishti AH, Li T (1997). Molecular characterization of abLIM, a novel actin-binding and double zinc finger protein. J Cell Biol.

[33] Li G, Huang S, Yang S, Wang J, Cao J, Czajkowsky DM (2018). abLIM1 constructs non-erythroid cortical actin networks to prevent mechanical tension-induced blebbing. Cell Discov.

[34] Schneider P, Bayo-Fina JM, Singh R, Kumar Dhanyamraju P, Holz P, Baier A (2015). Identification of a novel actin-dependent signal transducing module allows for the targeted degradation of GLI1. Nature Commun.

[35] Jin SH, Kim H, Gu DR, Park KH, Lee YR, Choi Y (2018). Actin-binding LIM protein 1 regulates receptor activator of NF-κB ligand-mediated osteoclast differentiation and motility. BMB Rep.

[36] Ma XL, Shen MN, Hu B, Wang BL, Yang WJ, Lv LH (2019). CD73 promotes hepatocellular carcinoma progression and metastasis via activating PI3K/AKT signaling by inducing Rap1-mediated membrane localization of P110β and predicts poor prognosis. J Hematol Oncol.

[37] Liu R, Zhou Z, Zhao D, Chen C (2011). The induction of KLF5 transcription factor by progesterone contributes to progesterone-induced breast cancer cell proliferation and dedifferentiation. Molecular endocrinology.

[38] Er EE, Valiente M, Ganesh K, Zou Y, Agrawal S, Hu J (2018). Pericyte-like spreading by disseminated cancer cells activates YAP and MRTF for metastatic colonization. Nat Cell Biol.

[39] Parmacek MS (2007). Myocardin-related transcription factors: critical coactivators regulating cardiovascular development and adaptation. Circ Res.

[40] Lundquist MR, Storaska AJ, Liu T-C, Larsen SD, Evans T, Neubig RR (2014). Redox modification of nuclear actin by MICAL-2 regulates SRF signaling. Cell.

[41] Pei H, Hu W, Guo Z, Chen H, Ma J, Mao W (2018). Long noncoding RNA CRYBG3 blocks cytokinesis by directly binding G-actin. Cancer Res.

[42] Potter CJ, Pedraza LG, Xu T (2002). Akt regulates growth by directly phosphorylating Tsc2. Nat Cell Biol.

[43] Wu C, Asokan SB, Berginski ME, Haynes EM, Sharpless NE, Griffith JD (2012). Arp2/3 is critical for lamellipodia and response to extracellular matrix cues but is dispensable for chemotaxis. Cell.

[44] Molinie N, Gautreau A (2018). The Arp2/3 regulatory system and its deregulation in cancer. Physiol Rev.

