# Supplementary material for: Rictor promotes cell migration and actin polymerization through regulating ABLIM1 phosphorylation in Hepatocellular Carcinoma
Source: Int J Biol Sci. 2020 Sep 1;16(15):2835–52. doi: 10.7150/ijbs.46285 (PMC7545703; doi:10.7150/ijbs.46285)
Supplement: Supplementary file 1 — Supplementary figures and tables. [file ijbsv16p2835s1.pdf]

FigureS1

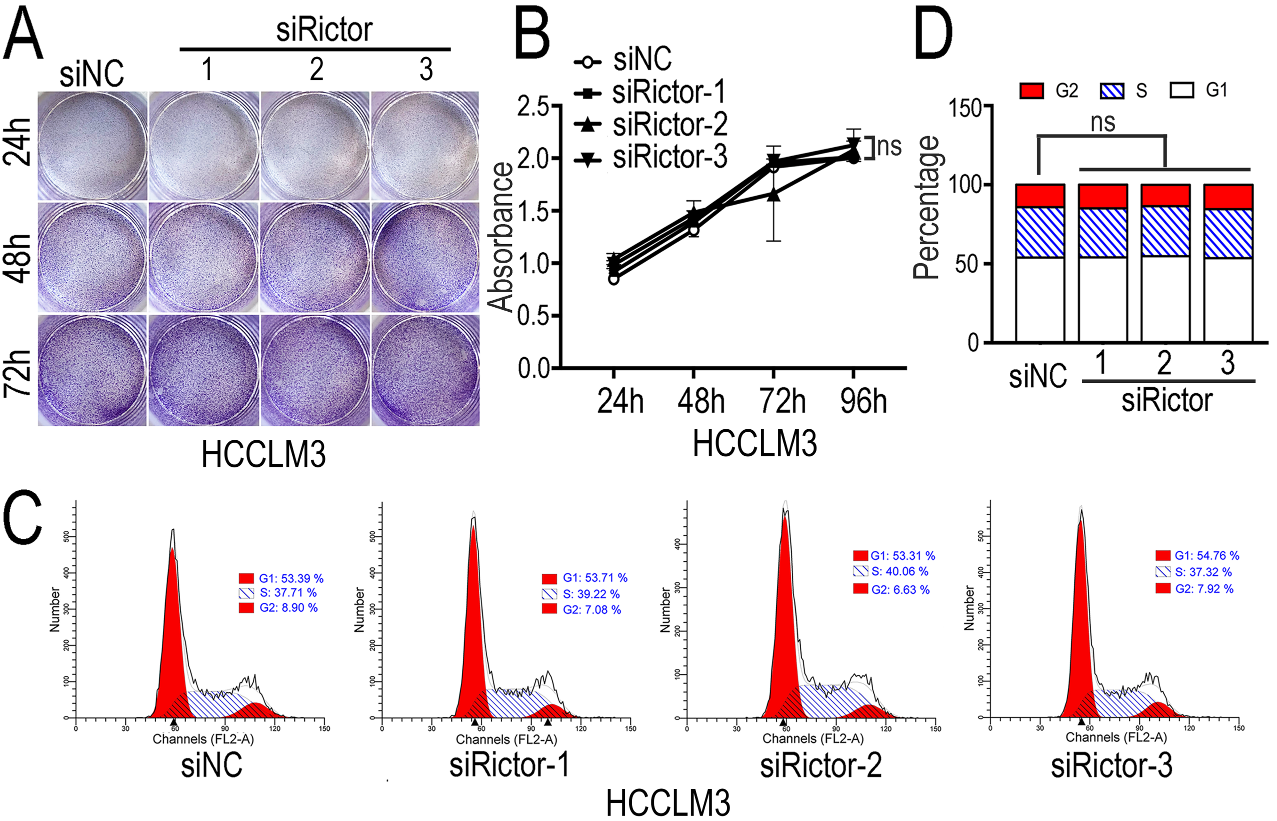
A
siRictor
siNC
1
2
3
24h
48h
72h
HCCLM3
B
siNC
siRictor-1
siRictor-2
siRictor-3
ns
Absorbance
2.5
2.0
1.5
1.0
0.5
0.0
24h
48h
72h
96h
HCCLM3
D
G2
S
G1
ns
Percentage
150
100
50
0
siNC
1
2
3
siRictor
C
G1: 53.39 %
S: 37.71 %
G2: 8.90 %
Number
Channels (FL2-A)
siNC
G1: 53.71 %
S: 39.22 %
G2: 7.08 %
Number
Channels (FL2-A)
siRictor-1
G1: 53.31 %
S: 40.06 %
G2: 6.63 %
Number
Channels (FL2-A)
siRictor-2
G1: 54.76 %
S: 37.32 %
G2: 7.92 %
Number
Channels (FL2-A)
siRictor-3
HCCLM3

FigureS2

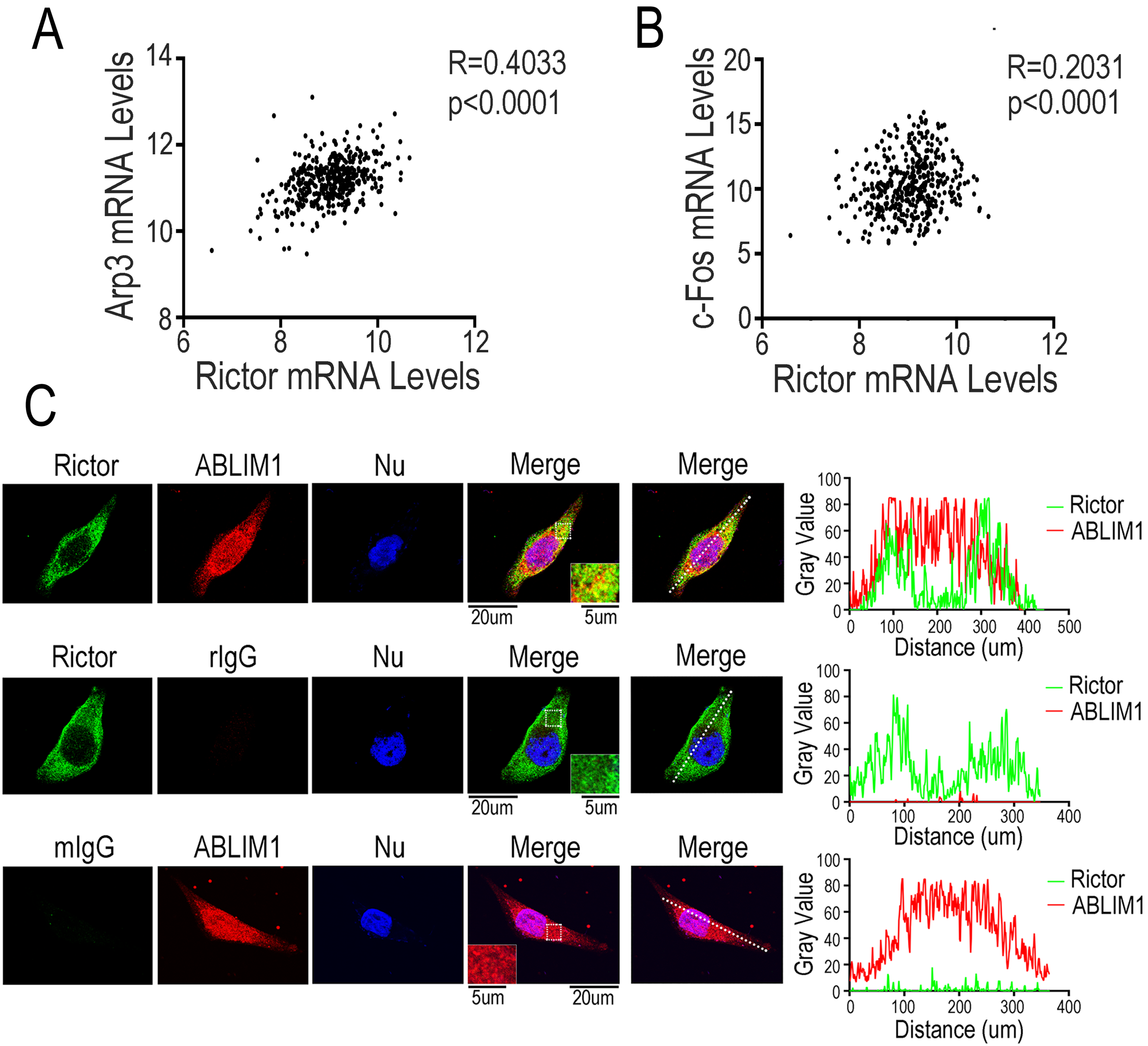
A
Arp3 mRNA Levels
Rictor mRNA Levels
R=0.4033
p<0.0001
B
c-Fos mRNA Levels
Rictor mRNA Levels
R=0.2031
p<0.0001
C
Rictor
ABLIM1
Nu
Merge
Merge
20um
5um
Gray Value
Distance (um)
Rictor
ABLIM1
Rictor
rIgG
Nu
Merge
Merge
20um
5um
Gray Value
Distance (um)
Rictor
ABLIM1
mIgG
ABLIM1
Nu
Merge
Merge
5um
20um
Gray Value
Distance (um)
Rictor
ABLIM1

FigureS3

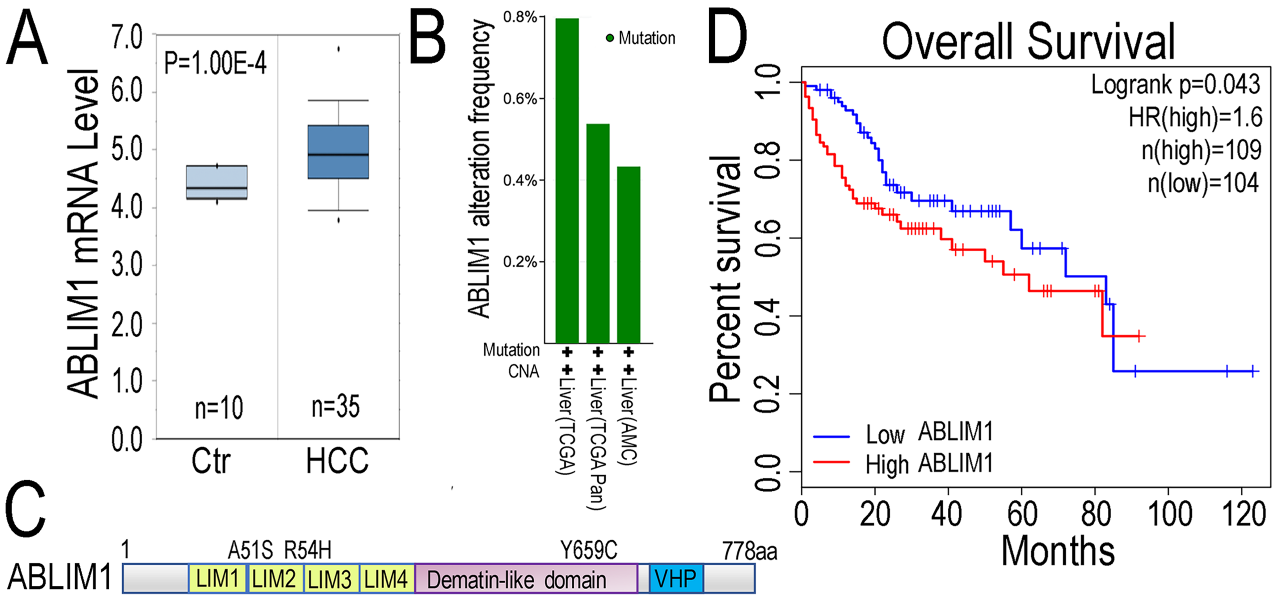
A
P=1.00E-4
ABLIM1 mRNA Level
7.0
6.0
5.0
4.0
3.0
2.0
1.0
0.0
n=10
n=35
Ctr
HCC
B
ABLIM1 alteration frequency
0.8%
0.6%
0.4%
0.2%
Mutation
Mutation
CNA
Liver (TCGA)
Liver (TCGA Pan)
Liver (AMC)
C
1
A51S R54H
Y659C
778aa
ABLIM1
LIM1
LIM2
LIM3
LIM4
Dematin-like domain
VHP
D
Overall Survival
Logrank p=0.043
HR(high)=1.6
n(high)=109
n(low)=104
Percent survival
1.0
0.8
0.6
0.4
0.2
0.0
Low ABLIM1
High ABLIM1
0
20
40
60
80
100
120
Months

Supplementary Figure Legends

Figure S1. Rictor does not affect the proliferation of HCC cells. (A) The HCCLM3 cells transfected with control (siNC) or three siRNAs against Rictor (siRictors) were seeded into 12-well plates and grown for 72 hrs. Cell colonies were stained with crystal violet after fixation with methanol. Representative images of stained cells in different groups are shown. **(B)** HCCLM3 cells transfected with siNC or siRictors were seeded into 96-well plates. After incubation of CCK-8 with cells at indicated time points, the absorbance at 450 nm was measured. Time course of cell growth in four groups was quantified from three independent experiments and growth curves were plotted with the means±SEM of absorbance versus time points. Statistical analysis was performed by Two-way ANOVA test with GraphPad Prism7. **(C)** The cell cycle was analyzed by flow cytometry in HCCLM3 cells transfected with siNC or siRictors at 72 hrs post-transfection. **(D)** Representative images were captured and the ratio of different phases during the cell cycle was analyzed by Two-way ANOVA test from three independent experiments.

Figure S2. Expression of Rictor is positively associated with expression of Arp3 or c-Fos in HCC tissues. (A)-(B) The expression correlation of Rictor and Arp3 or c-Fos was evaluated from the TCGA dataset. Relative mRNA levels of Rictor were respectively plotted against those of Arp3 or c-Fos. R represents a Pearson coefficient, and the p-value was shown. ***: $p<0.0001$; **: $p<0.01$. **(C)** HCCLM3 cells plated onto coverslips coated with fibronectin were stained with combinations of anti-ABLIM1 and/or anti-Rictor, or negative control (mouse IgG or rabbit IgG) antibodies, followed by incubation with Alexa488-conjugated and Alexa594-conjugated secondary antibodies. The nuclei were marked by DAPI staining. The stained images were visualized by confocal microscopy. The colocalization signals of Rictor and ABLIM1 in the dotted rectangle are enlarged. The distribution of both proteins on the white dotted line was analyzed among different groups by ImageJ software. Scale bar: 20um and 5um.

Figure S3. ABLIM1 is highly expressed in HCC tissues and correlated with poor prognosis. (A) The Oncomine database was utilized to analyze the ABLIM1 expression in HCC tissues (www.oncomine.org). Boxplots comparing ABLIM1 mRNA levels in HCC patients with those from non-tumor liver tissues are shown. Y-axis: ABLIM1 mRNA levels were expressed as log2 median-centered intensity. P-values were determined by Student's *t*-test. **(B)** The frequency of ABLIM1gene alteration in liver cancers was analyzed from the cBioPortal dataset. **(C)** Shown are somatic muations across the domains of ABLIM1 in liver cancer tissues. **(D)** Kaplan-Meier curves showing the overall survival analysis in HCC patients with high and low expression of ABLIM1 from the GEPIA datasets with Log-rank test p-values displayed.

Table S1. sgRNA, siRNA and primers sequences

**sgABLIM1 sequences**			
Target gene	sgRNA sequence	PAM	Strand
ABLIM1-1	CCAAGGACTACTGCCCACGT	GGG	-
ABLIM1-3	TAAGCAATCTACGAAGACCG	AGG	-

**ABLIM1 PCR primers sequences**			
Gene	Primer	Sequence	Notes
ABLIM1-1	Forward primer	TAGCTCCGTAGCTACACTT	PCR for ABLIM1 target region
	Reverse primer	GGAACAGAGAGAATTGTGG	
ABLIM1-3	Forward primer	CTAAGAAGCTGACTAGTAAGG	PCR for ABLIM1 target region
	Reverse primer	TTGGTGGTCTCTGTGGTCTA	
ABLIM1-3-OT1	Forward primer	AGGCTGGATGCCAGTTACT	ABLIM-1-sgRNA-3 off-target site 1
	Reverse primer	TCACCTGTACTGAGCCATG	
ABLIM1-3-OT2	Forward primer	AGCACTTCATGAGGCCAGG	ABLIM-1-sgRNA-3 off-target site 2
	Reverse primer	GGTTCAAGCAATTCTCCTG	
ABLIM1-3-OT3	Forward primer	TGGCATCAGTTAAGATTCTTG	ABLIM-1-sgRNA-3 off-target site 3
	Reverse primer	ACGTTCAGCTGGAATTCTGT	

**siRNA sequences**	
Gene	Sequence
siNC	UUCUCCGAACGUGUCACGUTT
siRictor-1	GCCUCCUGAAGCAUAAUCUUU
siRictor-2	CCCGGGACUUAUAAGUUAUUU
siRictor-3	CAACCCGGGACUUAUAAGUUU
siABLIM1-1	CUGCAACACUAGAUUCGAAUU
SiABLIM1-2	GUAGACUCCUUCGAACGUAUU

**Plasmid construction primers sequences**		
Gene	Primer	Sequence
Rictor-F1	Forward primer	ATGGCGGCGATCGGCCG
	Reverse primer	ACT CCT CAG TCA CAA CAG GT
Rictor-F2	Forward primer	ACCTGTTGTGACTGAGGAGT
	Reverse primer	TCCAGATGAAGCATTGAGCC
Rictor-F3	Forward primer	GGCTCAATGCTTCATCTGGA
	Reverse primer	ACTCGATGGCACAGATTCAC
Rictor-F4	Forward primer	GTGAATCTGTGCCATCGAGT
	Reverse primer	TTCATGAACCTGCTTGGTGT
Rictor-F5	Forward primer	ACACCAAGCAGGTTCATGAA
	Reverse primer	TCAGGATTCAGCAGATGTAT
ABLIM1-FL-WT	Forward primer	TTGGTACCGAGCTCGGATCC
	Reverse primer	ACTGTGCTGGATATCTGCAGAATTCT

ABLIM1-S214A	Forward primer	CAGCCGATGGCGTCCAGT
	Reverse primer	ACTGGACGCCATCGGCTG
ABLIM1-S214D	Forward primer	AGCCGATGGATTCCAGTCCG
	Reverse primer	CGGACTGGAATCCATCGGCT
ABLIM1-S431A	Forward primer	GAGGACTTTGGCTCCTACTCCAT
	Reverse primer	ATGGAGTAGGAGCCAAAGTCCTC
ABLIM1-S431D	Forward primer	CGAGGACTTTGGATCCTACTCCAT
	Reverse primer	ATGGAGTAGGATCCAAAGTCCTCG
MKL1-F1	Forward primer	CTGTACAAGGGATCCGAATTCGTGGATTTCTCCAGTGTGGT
	Reverse primer	GCAGACTTGGGTTGGCTTTG
MKL1-F2	Forward primer	CAAAGCCAACCCAAGTCTGC
	Reverse primer	AGGGTCTATGTGGTTGGTGG
MKL1-F3	Forward primer	CCACCAACCACATAGACCCT
	Reverse primer	GCTCTCGCTGAATTGGCGGCCGCCTACAAGCAGGAATCCCAGT
**Real-time quantitative PCR primers sequences**		
Gene	Primer	Sequence
Rictor	Forward primer	TGGATCTGACCCGAGAACCT
	Reverse primer	TCCTCATAGTGAAAGCCCAGT
ABLIM1	Forward primer	AAATCCATGCCAAGATGCCT
	Reverse primer	CTCCGGTCCTCAACAATCAG
GAPDA	Forward primer	GAGTCAACGGATTTGGTCGT
	Reverse primer	TTGATTTTGGAGGGATCTCG
c-Fos	Forward primer	GACTGATACACTCCAAGCGG
	Reverse primer	GGTCATCAGGGATCTTGCAG
Arp3	Forward primer	ATTCAAGCACCAACATTCACT
	Reverse primer	AATGCTTCTGGGATTGAAGGC

Table S2.

Peptide Sequences	Protein Accessions	Gene Symbol	Description	Phospho sites	$Log_2(Ratio_{KD/C})$	p value
**downregulated**						
SHTSEGAHLDITPNSGAAGNSAGPK	E5RJY1	NDRG1	Protein NDRG1	T3	-5.08	0.05
KKSSSDPGIPGGPQAIPATNSPDHSDHTLSVSSDSGHSTASAR	E9PCX8	TNS3	Tensin-3	S3	-4.67	0.03
SRTSVQTEDDQLIAGQSAR	Q8N1C0	CTNNA1	Cadherin-associated protein, alpha 1	S4	-4.39	0.04
KKLGAGEGGEASVSPEKTSTTSK	J3KQ96	TCOF1	Treacle protein (Fragment)	S14	-2.79	0.04
SEPVINNDNPLESNDEKEGQEATCSRPQIVPEPMDFR	P23497	SP100	Nuclear autoantigen Sp-100	S13	-2.62	0.02
ETNLDSLPLVDTHSKR	P08670	VIM	Vimentin	T2	-2.42	0.05
LKSEDGVEGDLGETQSR	Q09666	AHNAK	Neuroblast differentiation-associated protein AHNAK	S3	-2.37	0.004
KLGDVSPTQIDVSQFGSFKEDTK	P46821	MAP1B	Microtubule-associated protein 1B	S6	-2.35	0.04
TELSPSFINPNPLEWFASEEPTEESEKPLTQSGGAPPPPGGK	P46821	MAP1B	Microtubule-associated protein 1B	S4	-2.32	0.03
SKGHYEVTGSDDETGKLQGSGVSLASK	Q09666	AHNAK	Neuroblast differentiation-associated protein AHNAK	S10	-2.32	0.03
EATSDPSRTPEEEPLNLEGLVAHR	E9PGF5	TNS1	Tensin-1	T9	-2.30	0.03
RSSDTSGSPATPLK	Q7Z5R6	APBB1IP	Amyloid beta A4 precursor protein-binding family B member 1-interacting protein -	S3	-2.27	0.05
MVIQGPSSPQGEAMVTDVLEDQKEGR	A6NNK5	TP53BP1	Tumor suppressor p53-binding protein 1	S8	-2.19	0.05
QLHLEGASLELS	F8VTL3	MYH10	Myosin-10	S12	-2.14	0.05

DDDTESKTSDV NETQPPQSE						
LKATVTPSPVKG K	Q9H1E3	NUCKS1	Nuclear ubiquitous casein and cyclin-dependent kinase substrate 1	T4; S8	-2.02	0.04
ADSRESLKPAAK PLPSK	P46821	MAP1B	Microtubule-associat ed protein 1B	S3	-2.00	0.01
THSFENVSCHLP DSR	B7Z660	DENND4 C	DENN domain-containing protein 4C	S3	-1.99	0.03
RKASPEPPDSAE GALKLGEEQQR	Q9H1B7	IRF2BPL	Interferon regulatory factor 2-binding protein-like	S4	-1.98	0.04
GHYEVTGSDDE TGKLQGSGVSL ASKK	Q09666	AHNAK	Neuroblast differentiation-associ ated protein AHNAK	S8	-1.97	0.01
RLSSLRASTSKS ESSQK	A2A3R5	RPS6	40S ribosomal protein S6	S4; S8	-1.94	0.05
KASPEPPDSAEG ALKLGEEQQR	Q9H1B7	IRF2BPL	Interferon regulatory factor 2-binding protein-like	S3	-1.92	0.02
VASGEQKEDQSE DKKRPSLPSSPSP GLPK	O43294	TGFB1I1	Isoform 2 of Transforming growth factor beta-1-induced transcript 1 protein	S18; S21	-1.89	0.05
MDSDEDEKEGE EEKVAKR	Q96ST2	IWS1	Protein IWS1 homolog	S3	-1.86	0.04
SKGHYEVTGSD DETGKLQGSGV SLASKK	Q09666	AHNAK	Neuroblast differentiation-associ ated protein AHNAK	S10	-1.82	<0.001
DCLCQLCAQPM SSSPKETTFSSNC AGCGR	H0Y3K7	ABLIM1	Actin-binding LIM protein 1 (Fragment)	S12	-1.77	0.02
EKEDTDVADGC RETPTKTLEGDG DQER	Q8IVF2	AHNAK2	Isoform 3 of Protein AHNAK2	T14	-1.64	0.04
SQSTTFNPDDMS EPEFK	Q86W92	PPFIBP1	Isoform 2 of Liprin-beta-1	S3	-1.63	0.03
SQSTTFNPDDMS EPEFKR	Q86W92	PPFIBP1	Isoform 2 of Liprin-beta-1	S3	-1.56	0.02
DAHDVSPTSTDT EAQLTVERQEQK	Q8IVF2	AHNAK2	Isoform 3 of Protein AHNAK2	S6	-1.51	0.01
KGDRSPEPGQT	Q09666	AHNAK	Neuroblast	S5	-1.41	0.05

WTR			differentiation-associated protein			
HRAEAPPLEREDSGTFSLGK	G5E9Q4	PRKRA	Interferon-inducible double stranded RNA-dependent protein kinase activator A	S13	-1.33	0.04
DRASPAAAEEVVPEWASCLK	Q8N3V7-2	SYNPO	Isoform 2 of Synaptopodin	S4	-1.22	0.05
RAASSDQLRDNSPPPAFKPEPPKAK	F5H301	TJP2	Tight junction protein ZO-2	S5; S12	-1.22	0.02
TASFSESRADEVAPAKK	P53396-2	ACLY	Isoform 2 of ATP-citrate synthase	S3	-1.18	0.03
SVGKVEPSSQSPGRSPR	K7EKH8	ERC1	ELKS/Rab6-interacting/CAST family member 1 (Fragment)	S15	-1.18	0.04
SQDATFSPGSEQAEKSPGPIVSR	C9J0I9	ZC3HC1	Nuclear-interacting partner of ALK	S16	-1.18	0.003
TNTMNGSKSPVISRPK	Q8N8S7-2	ENAH	Isoform 2 of Protein enabled homolog	S9	-1.17	0.02
EAEALLQSMGLTPESPIVPPPMSPSSK	E7EV09	DYNC1I2	Cytoplasmic dynein 1 intermediate chain 2 (Fragment)	S26	-1.13	0.03
GGNVFAALIQDQSEEEEEEEKHPPKPAKPEK	Q8NE71	ABCF1	ATP-binding cassette sub-family F member 1	S13	-1.12	0.03
REFITGDVEPTDAESEWHSENEEEEKLAGDMK	C9JZI7	NAP1L4	Nucleosome assembly protein 1-like 4 (Fragment)	S19	-1.05	0.04
HYEDGYPGGSDNYGSLSR	O60716-21	CTNND1	Isoform 3A of Catenin delta-1	S15	-0.91	0.03
RASVCAEAYNPDEEEDDAESR	P31323	PRKAR2B	cAMP-dependent protein kinase type II-beta regulatory subunit	S3	-0.90	0.022511
EALREHSNPSPSQDTDGTK	O60292	SIPA1L3	Signal-induced proliferation-associated 1-like protein 3	S10	-0.87	0.04
GSYGSDAEEEEYRQQLSEHSKR	F5H301	TJP2	Tight junction protein ZO-2	Y3	-0.82	0.006
DSQDASAEQSDHDDEVASLASASGGFGTK	Q6P2E9	EDC4	Isoform 2 of Enhancer of mRNA-decapping	S6	-0.81	0.05

			protein 4			
TQSSSCEDLPSTTQPK	O14936	CASK	Isoform 5 of Peripheral plasma membrane protein CASK	S5	-0.81	0.03
EFITGDVEPTDAESEWHSENEEEEKLAGDMK	C9JZI7	NAP1L4	Nucleosome assembly protein 1-like 4 (Fragment)	S18	-0.80	0.04
NKDSGSDTASAIIPSTTPSVDSDDESVVKDK	Q6WKZ4	RAB11FIP1	Isoform 4 of Rab11 family-interacting protein 1	S22	-0.79	0.05
KVEEEGSPGDPDHEASTQGR	Q9NZT2	OGFR	Isoform 2 of Opioid growth factor receptor	S7	-0.72	0.04
RPDPDSDEDEDYERER	Q5W011	RBM17	RNA binding motif protein 17 (Fragment)	S6	-0.70	0.05
QLSSGVSEIR	P04792	HSPB1	Heat shock protein beta-1	S3	-0.68	0.05
KGAGDGSDEEVDGKADGAEAKPAE	P35579	MYH9	Myosin-9	S7	-0.69	0.05
TLSPTPSAEGYQDVRDR	H0Y3K7	ABLIM1	Actin-binding LIM protein 1 (Fragment)	S3	-0.689	<0.001
SLYASSPGGVYATR	P08670	VIM	Vimentin	S6	-0.67	0.03
HKYVSGSSPDLVTR	Q15678	PTPN14	Tyrosine-protein phosphatase non-receptor type 14	S7	-0.63	0.03
**upregulated**						
AKTQTPPVSPAPQPTEERLPSSPVYEDAASFK	Q14247	CTTN	Src substrate cortactin	T3	0.65	0.04
HIKEEPLSEEEPCTSTAIASPEK	Q9Y2X3	NOP58	Nucleolar protein 58	S8	0.69	0.03
SQSPAASDCSSSSSSASLPSSGR	O95817	BAG3	BAG family molecular chaperone regulator 3	S3	0.83	0.04
ATNESEDEIPQLVPIGKK	O76021	RSL1D1	Ribosomal L1 domain-containing protein 1	S5	0.86	0.03
FEEESKEPVADEEEDSDDDVEPI	P54105	CLNS1A	Methylosome subunit pICln	S17	0.91	0.05

TEFR						
HIKEEPLSEEEPCTSTAIASPEKK	Q9Y2X3	NOP58	Nucleolar protein 58	S8	1.00	0.005
QSFDDNDSEELEDKDSK	O60841	EIF5B	Eukaryotic translation initiation factor 5B	S8	1.02	0.03
DYDDMSPR	P61978	HNRNPK	Heterogeneous nuclear ribonucleoprotein K	S6	1.04	0.05
CSDVSELSSSPPGPYHQEPYVCKPEER	F5H610	PRKAB1	5'-AMP-activated protein kinase subunit beta-1 (Fragment)	S10	1.05	0.01
KIALESEGRPEEQMESDNCSGGDDDWTHLSSK	Q13501	SQSTM1	Sequestosome-1	S20	1.20	0.04
HVDSLSQRSPK	Q9UHD8	41526	Septin-9	S6	1.28	0.05
AEEDEILNRSPR	B4E2T8	CANX	Calnexin	S10	1.38	0.05
YGPADVEDTTGSGATDSKDDDDIDLFGSDDEEESEEAKRLR	P24534	EEF1B2	Elongation factor 1-beta	S28	1.47	0.03
NTPSQHSHSIQHSPER	Q9NYF8-3	BCLAF1	Isoform 3 of Bcl-2-associated transcription factor 1	S13	1.56	0.01
SSVQGASSREGSPARSSTPLHSPSPIR	O95817	BAG3	BAG family molecular chaperone regulator 3	S8; S12	1.63	0.03
MPQDGSDDEDEEWPTLEK	F8WBL2	BYSL	Bystin	S6	1.82	0.04
AADPPAENSSAPEAEQGGAE	P67809	YBX1	Nuclease-sensitive element-binding protein 1	S10	1.92	0.04
MDAPASGSACSGLNKQEPGGSHGSETEDTGR	Q9UKJ3-2	GPATCH8	Isoform 2 of G patch domain-containing protein 8	S24	1.93	0.02
TMFAQVESDDEEAKNEPEWK	Q9NW82	WDR70	WD repeat-containing protein 70	S8	2.25	0.01
QGPVSQSATQQPVTADKQQGHEPVSPR	Q9BTA9-5	WAC	Isoform 4 of WW domain-containing adapter protein with coiled-coil	S25	2.34	0.02
LKSPSQKQDGGTAPVASASPK	Q96T37-2	RBM15	Isoform 2 of Putative RNA-binding protein	S3	2.81	0.04

			15			
HAPSPEPAVQGTGVAGVPEESGDAAAIPAK	P13051	UNG	Uracil-DNA glycosylase	S4	3.13	0.03
SGSMDPSGAHPSVR	Q07666-3	KHDRBS1	Isoform 3 of KH domain-containing, RNA-binding, signal transduction-associated protein 1	S3	3.68	0.06
